# Team Synergies in Professional Football: A Goal-Referenced Performance Indicator for Rapid Ball Recovery in Negative Transitions

**DOI:** 10.3390/sports14090374

**Published:** 2026-09-01

**Authors:** Enrico Serra, Daniel Carrilho, Li Xiao, Cristian Savoia, Marco Beato, Silvia Coppola, Rodolfo Vastola, Duarte Araújo

**Affiliations:** 1 Department of Neurosciences, Biomedicine and Movement Sciences, University of Verona, 37124 Verona, Italy; 2 Department of Human, Philosophical and Educational Sciences, University of Salerno, 84084 Fisciano, Italy; sicoppola@unisa.it; 3 CIPER, Faculdade de Motricidade Humana, Universidade de Lisboa, 1495-751 Oeiras, Portugal; danielcarrilho@fmh.ulisboa.pt (D.C.); xiao.li@edu.ulisboa.pt (L.X.); daraujo@fmh.ulisboa.pt (D.A.); 4 The Research Institute for Sport and Exercise Sciences, The Tom Reilly Building, Liverpool John Moores University, Liverpool L3 5AH, UK; cristiansavoia@gmail.com; 5 Department of Education and Sport Sciences, Pegaso Telematic University, 80143 Naples, Italy; marcobeato.coach@gmail.com; 6 Department of Political and Social Studies, University of Salerno, 84084 Fisciano, Italy; rvastola@unisa.it

**Keywords:** ecological dynamics, GNSS tracking, angular concentration, directional coherence, tactical coordination, out-of-possession phase, performance analysis, team monitoring, soccer

## Abstract

Negative transitions require teams to reorganise rapidly from attack to defence, yet few positional indicators describe collective reorganisation in relation to possession recovery. This study examined whether rβ, a GNSS-derived angular concentration performance indicator referenced to the goal to be protected, was associated with controlled possession recovery within 10 s after a negative transition. In this retrospective observational study, five official matches of a single professional team (Serie A and UEFA Conference League) were analysed. Negative-transition episodes ending with controlled recovery were classified as FAST (≤10 s) or SLOW (>10 s). For each outfield player, the angular deviation between movement direction and the direction to the protected goal was computed; rβ was calculated at the team level using Kuramoto’s order parameter. The sample comprised 248 episodes (133 FAST; 115 SLOW). FAST episodes showed higher mean rβ values than SLOW episodes (0.717 vs. 0.600; difference = 0.117; 95% CI [0.085, 0.150]; *p* < 0.001; and *d* = 0.906). This pattern remained consistent across thresholds, fixed-window analyses, zones, and matches. Among recovered transitions, greater goal-referenced angular concentration was associated with controlled recovery within 10 s; rβ may serve as a process indicator of immediate defensive reorganisation that could support training, team monitoring and performance analysis.

## 1. Introduction

A football match can be interpreted as a complex and adaptive system, in which performance emerges from the continuous interaction between players, the ball and space [1,2,3]. In match analysis, focusing solely on individual performance or on discrete events risks failing to capture the relational nature of the game, in which players’ actions are continuously regulated by the information available in the performance environment [4,5,6]. As has been argued, ecological dynamics is a theoretical framework that can guide performance analysis as a situated, dynamic and non-linear process, regulated by individual, environmental and task constraints [5,6,7].

This perspective is particularly relevant for the defensive phase of the match, in which a team is out of possession of the ball and tries to recover it or to prevent the team in possession from progressing towards the goal to score. In the defensive phase, team effectiveness depends on the spatio-temporal coordination between players (i.e., defenders), particularly on their ability to maintain adaptive collective organisation during the out-of-possession phase [8,9]. In team sports, team coordination has been interpreted through the concept of team synergies, that is, functional intra-team couplings through which players collectively regulate their actions to act on shared affordances (to achieve team goals [10,11,12]).

The development of positional tracking systems has made it possible to quantify collective tactical behaviour through variables such as length, width, occupied surface area, stretch index, centroids and interpersonal distances [13,14,15]. Although useful, these metrics mainly describe team shape, dispersion or co-movement and do not always clarify the functional meaning of collective behaviour in relation to specific defensive problems, i.e., they fail to capture the team–environment link [12,16]. In line with this limitation, a recent scoping review on defensive synergies in football highlighted that the link between collective team properties and defensive outcomes remains insufficiently systematised [17] (submitted for publication).

A possible step forward is to test collective indicators anchored to ecologically meaningful references of the defensive task (what is termed eco-physical variables [18]) and, specifically for the purposes of this paper, verifying their association with observable outcomes of the out-of-possession phase. Negative transitions (i.e., transitions from the attacking to the defensive phase) represent a particularly suitable context for examining this relationship because, after losing possession, the team must rapidly reorganise from an attacking structure into a coordinated defensive response [19,20]. In this phase of instability and time pressure, rapid ball recovery can be considered a relevant process indicator of immediate defensive reorganisation [19,20]. Within this framework, positional data derived from Global Navigation Satellite System (GNSS) technologies are now routinely collected by professional clubs and used by coaches, performance analysts and sport scientists to monitor players and to inform their decisions. Although these systems are used mainly for physical and external-load monitoring, the same continuous tracking of players’ coordinates also offers a concrete possibility for deriving interpretable collective tactical indicators, thereby extending an established monitoring technology from the physical domain to the analysis of collective behaviour on the pitch [13]. Therefore, the aim of the present study is to examine whether a team performance indicator of players’ directional coherence relative to the goal to be protected (rβ), computed from GNSS positional data, is associated with the speed of controlled possession recovery after negative transitions in professional football. It is hypothesised that, among episodes ending with valid controlled recovery, those resolved within the predefined 10 s temporal threshold will show higher rβ values than later episodes and that higher rβ values are associated with a greater probability of rapid recovery. Establishing this association would also be of practical interest: it would make the collective organisation of the defensive phase observable through a single, interpretable indicator, one that could inform performance analysis and training in professional football.

## 2. Materials and Methods

### 2.1. Study Design and Sample

The present observational study analysed five official matches of a team competing in the 2024/2025 season of the highest level of competition in Italy (Serie A; *n* = 3) and in European competition (UEFA Conference League; *n* = 2). The analysed team maintained the same reference playing system, coded as 1-3-4-2-1, and the sample comprised three home matches and two away matches, each against a different opponent. Study reporting was guided by the STROBE checklist for observational studies [21]. The sample size was determined by the availability of official matches with synchronisable GNSS and video data and by the number of episodes meeting the inclusion criteria; these matches, played at the start of the season, constituted a convenience sample. Given the observational nature of the study and the restricted access to professional data, no a priori sample size calculation was performed. Episodes were identified by applying predefined operational criteria to the available sequences.

The units of analysis were the 248 negative-transition episodes that met the predefined operational criteria and ended with valid controlled possession recovery by the analysed team, distributed across the five analysed matches (Match 1 = 54, Match 2 = 44, Match 3 = 59, Match 4 = 49, and Match 5 = 42). Therefore, the results refer to recovery speed among recovered episodes, not to the overall ability to regain the ball after every negative transition.

### 2.2. Positional and Video Data

Player positional data were provided by K-Sport and acquired using the K-AI GNSS tracking system (K-Sport World S.R.L., Pesaro, Italy). The system integrates multi-constellation satellite tracking (GPS, GLONASS, Galileo, and BeiDou) with a synchronised inertial measurement unit (IMU), including a triaxial accelerometer (±16 g), gyroscope (±2000°·s^−1^) and magnetometer sampled at 40 Hz. Original data were captured with a frequency of 50 Hz and provided by the company in an already preprocessed format sampled at 10 Hz. As with any GNSS tracking, the positional signal carries a small measurement error; its influence on the heading angle was mitigated by the low-speed handling and examined in the sensitivity analyses (Supplementary Methods S1). In the final reference system, the x-axis represented the longitudinal dimension of the pitch and the y-axis the lateral dimension. Coordinates were oriented so that the goal to be defended by the analysed team maintained the same spatial reference across all sequences. The data provider had no role in the study design, episode selection, analysis or interpretation.

Matches were also analysed using official video recordings from the match venue at 25 frames per second, which were used to verify negative-transition episodes and the moment of possession recovery. An ad hoc script synchronised the positional time series with the video. For further verification, three independent UEFA A coaches, with 24.5 ± 2.1 years of experience, reviewed the full match videos by applying the same operational criteria as the main coder. The observers were blinded to rβ values and to the variables derived from recovery time and to the study hypothesis. Agreement was assessed at the episode level, considering sequence eligibility, identification of the negative transition, controlled possession by the opponent and controlled possession recovery by the analysed team. All included episodes were identified by the observers as valid negative transitions ending with controlled recovery, with no discrepancies compared with the main coder; therefore, inter-observer episode-level agreement for event validity and identity was perfect (100%).

### 2.3. Episode Identification and Outcome Definition

In the present study, a negative transition began when the opposing team acquired effective and controlled possession after a loss of possession by the analysed team. Controlled possession was identified when one player made at least two consecutive controlled touches, or when two players from the same team made two consecutive controlled touches without a controlled intervention by the opponent [22,23]. The initial instant of the episode, corresponding to the operational time of possession loss by the analysed team ${(t}_{l})$, was assigned to the first controlled touch by the opposing team, provided that the controlled possession criterion was retrospectively satisfied. Episodes developed within the dynamic flow of play and ending with controlled possession recovery by the analysed team were included [24]. Sequences starting directly from goal kicks, free kicks or corner kicks; uncontrolled changes of possession such as deflections, rebounds, tackles or accidental touches; and sequences ending with a shot, foul or interruption of play were excluded [20,25]. In the case of sequences following a throw-in, the episode was defined from the first controlled possession on the pitch. The criteria were applied before the calculation of the derived variables. Across the five matches, 345 negative-transition sequences were identified through video observation; 97 were excluded on the basis of these criteria, yielding the 248 episodes analysed.

**Figure 1:**
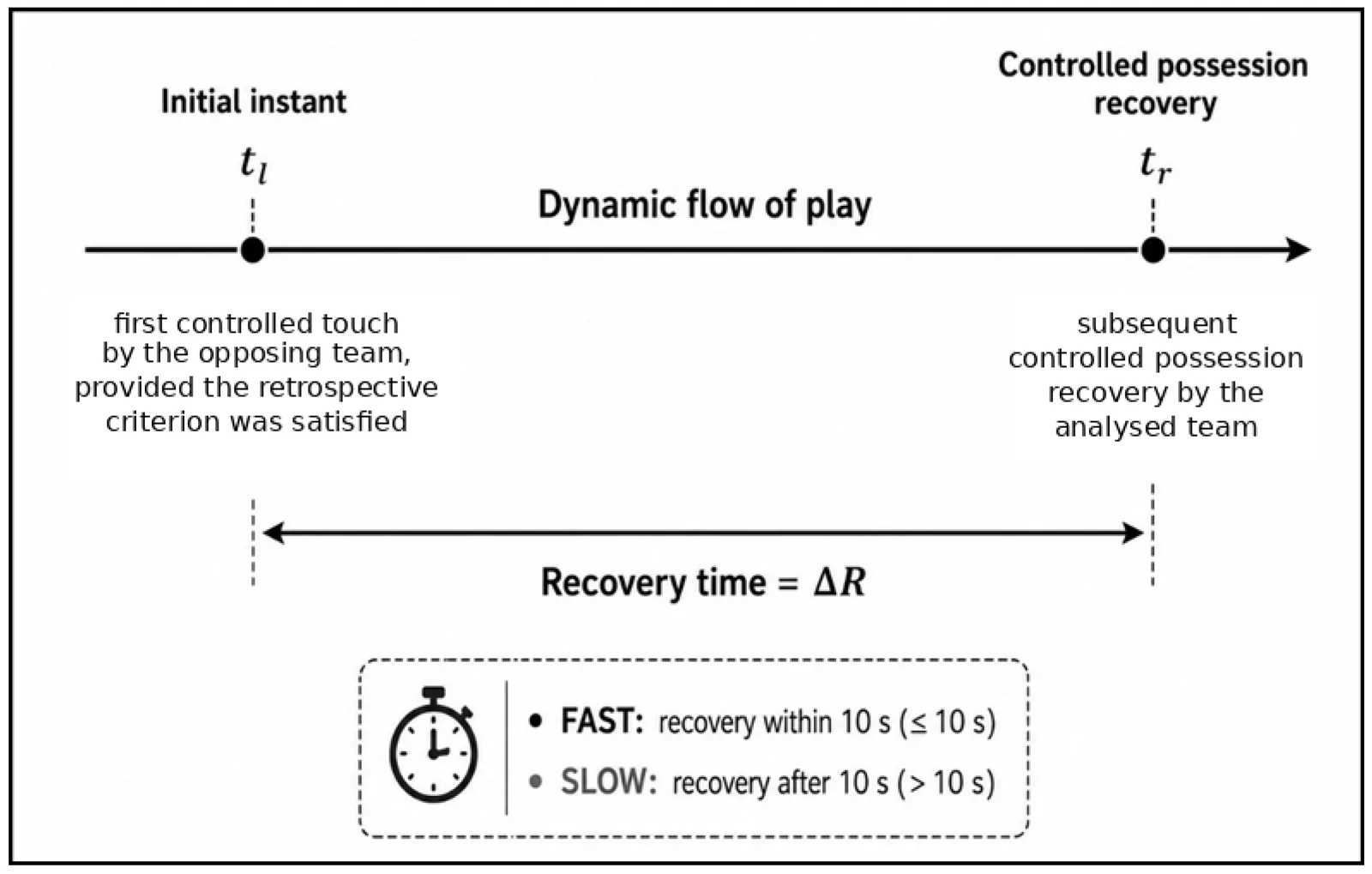
Schematic representation of recovery time during negative transitions. The initial instant of the episode was defined as the first controlled touch by the opposing team, provided that the retrospective criterion was satisfied. Recovery time (Δ*R*) was calculated as the interval between the initial instant (*t*_l_) and the subsequent controlled possession recovery by the analysed team (*t*_r_). Episodes were classified as FAST when Δ*R* ≤ 10 s and SLOW when Δ*R* > 10 s. Only outfield players were considered.

Recovery time (ΔR) was calculated as the interval, in seconds, between $t_{l}$ and the subsequent controlled possession recovery ($t_{r}$) by the analysed team, applying the same operational criterion used for possession acquisition. Episodes were classified as FAST when recovery occurred within 10 s (≤10 s) and as SLOW when recovery occurred after 10 s (>10 s). The 10 s threshold was adopted a priori as an operational reference to identify rapid recoveries, in continuity with the concept of defensive reaction time proposed by Vogelbein et al. [19]. Across the included episodes, 133 were classified as FAST and 115 as SLOW (53.6% and 46.4%, respectively), with a mean possession recovery time of 13.38 s (SD = 11.31 s). Median recovery time was 5.6 s (IQR 4.0–7.3; range 1.0–10.0) in FAST episodes and 18.8 s (IQR 14.5–26.9; range 10.2–59.7) in SLOW episodes. In all analyses, only outfield players were considered, excluding the goalkeeper (Figure 1).

### 2.4. Computation of rβ

**Figure 2:**
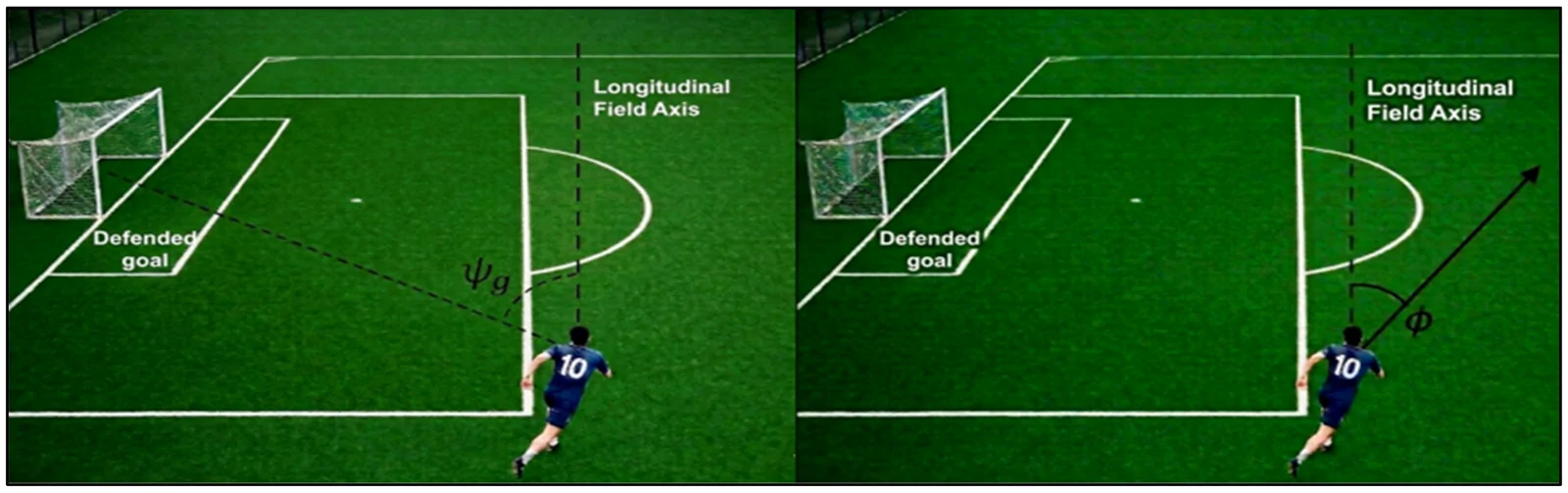
Schematic representation of the angular variables used in the analysis. The goal angle (ψg) represents the direction from the player to the centre of the defended goal, whereas the heading angle (φ) represents the instantaneous direction of player movement relative to the longitudinal axis of the field.

For each player and at each time instant, three angles were calculated: the goal angle (ψg), defined as the direction from the player towards the centre of the goal to be defended; the heading angle (φ), defined as the player’s instantaneous direction of movement (Figure 2); and the error angle (β), defined as the deviation between the player’s direction of movement and the line towards the goal to be defended.

For the calculation of φ, the components of the velocity vector were derived through numerical differentiation of the x and y coordinates, in line with procedures applied to football tracking data [26]. Since at very low speeds the direction of the velocity vector is more sensitive to measurement noise [27], samples with speed < 0.35 m/s were considered poorly informative samples, and not missing data, for the estimation of φ and were circularly interpolated between adjacent valid values, avoiding artificial discontinuities between −π and +π. No included episode had insufficient GNSS/video data for synchronisation or calculation of rβ. Technical checks with alternative speed thresholds and alternative procedures for handling low-speed samples are described in Supplementary Methods S1 and summarised in Supplementary Table S1. For each player, β was calculated as the circular difference between φ and ψg, reported within the interval [−π, +π]. The sign convention adopted was φ − ψg. This convention does not modify the value of rβ, because the order parameter used is based on the modulus of the mean complex vector.

The procedure follows the logic of the ψg–φ–β model proposed by López-Felip et al. [16], adapting it to the defensive context through the use of the goal to be protected as the angular reference. In the present study, cluster phase analysis (CPA) was not applied as a complete framework for analysing the synchronisation of displacement time series. The analysis focused on Kuramoto’s order parameter [28], used to summarise the concentration of a task-specific circular variable, β, among outfield players. Since β was already defined as a goal-referenced angular deviation between instantaneous direction of movement and the goal to be protected, it was not necessary to extract continuous phases through the Hilbert Transform from the longitudinal and lateral displacement time series. The β values of the players considered were therefore summarised using Kuramoto’s order parameter. For each time instant, rβ was calculated as

rβ(*t*) = |(1/*N*) Σ*_i_*_= 1_*^N^* e*^j^*^β^*^i^*^(*t*)^| (1)

where *N* represents the number of outfield players included in the calculation at time *t*, *j* represents the imaginary unit (*j*^2^ = −1), and β*_i_*(*t*) represents the β value of player *i* at time *t*. As a reference for interpreting this scale, the value of rβ expected for *N* = 10 uniformly random headings is ≈0.28 (chance baseline), whereas rβ approaches 1 as headings become perfectly aligned. This modulus is invariant to the common orientation of the β values so that a coordinated retreat toward the defended goal (β ≈ 0) and coordinated movement away from it (β ≈ π) yield the same rβ. For this reason we also computed the mean resultant direction, $\bar{\psi \beta }$ = arg(Σ*_i_* e*^j^*^β*i*^), with 0° denoting collective movement toward the goal to be defended.

**Figure 3:**
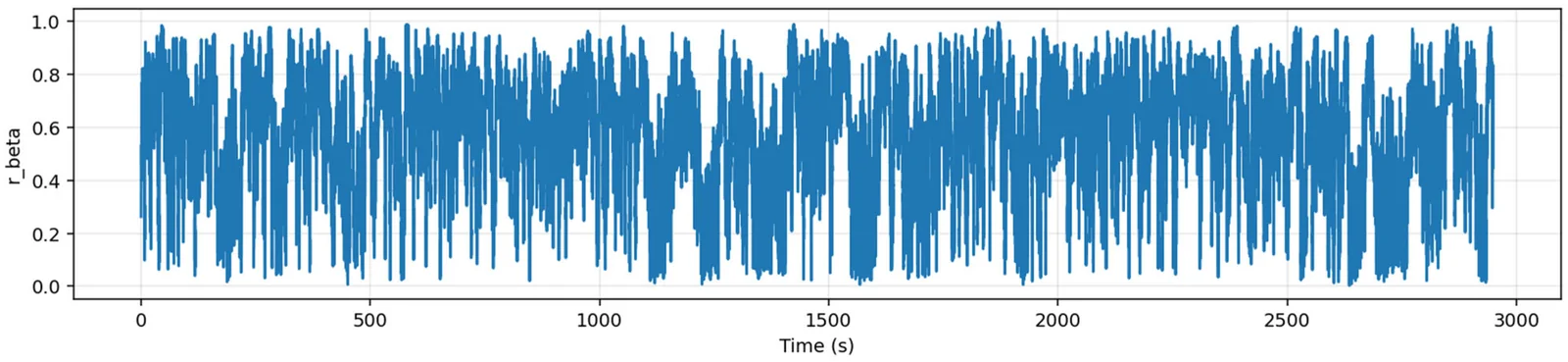
Example of the continuous rβ signal during the first half of a match included in the study. The instantaneous signal fluctuates markedly from frame to frame, which motivates the time-averaging of rβ over the analysed windows.

In all included episodes, the calculation was performed on the 10 outfield players of the analysed team present on the pitch; any substitutions between episodes did not modify *N*. The rβ index ranges between 0 and 1: values close to 0 indicate a more dispersed distribution of β among players, whereas values close to 1 indicate lower angular dispersion and, operationally, greater collective coherence of movement directions relative to the angular reference of the goal to be protected (Figure 3).

### 2.5. Time-Windowing Strategy and Sensitivity Analyses

The main analysis was based on the predefined 10 s threshold. For each episode, rβ was averaged over the observed recovery window. In FAST episodes, this window extended from $t_{l}$ to $t_{r}$, because recovery occurred within 10 s. In SLOW episodes, the window extended from $t_{l}$ to 10 s after $t_{l}$. The duration of this window therefore differed between groups by construction, being equal to recovery time in FAST episodes and fixed at 10 s in SLOW episodes. Since the signal was sampled at regular intervals of approximately 0.1 s, this metric represented the time-averaged mean value of rβ within the analysed window. The primary metric was named Mean_0_10_obs, indicating that rβ was averaged within the observed recovery window, capped at 10 s.

Sensitivity analyses were conducted using two complementary approaches. First, the observed-window approach was repeated using alternative thresholds of 8, 12 and 15 s. For each threshold, episodes were reclassified as FAST or SLOW, and rβ was averaged within the corresponding observed analysis window: from $t_{l}$ to $t_{r}$ for FAST episodes, and from $t_{l}$ to the selected threshold for SLOW episodes. These analyses tested whether the results were robust to the choice of temporal threshold.

Second, fixed-window landmark analyses were conducted over the first 2 s, 3 s and 5 s after $t_{l}$. In these analyses, rβ was calculated over identical temporal windows for all episodes, while the FAST/SLOW classification remained based on the original 10 s threshold. For each landmark analysis, only episodes still ongoing at the end of the fixed window were included. Episodes already recovered before the landmark endpoint were excluded from that specific analysis to avoid post-recovery samples and to ensure complete and comparable rβ windows.

For example, in the 0–2 s landmark analysis, rβ was calculated only for episodes that were still ongoing at 2 s. These episodes were then classified as FAST if recovery occurred after 2 s but within 10 s and as SLOW if recovery occurred after 10 s. The same logic was applied to the 0–3 s and 0–5 s landmarks. In this way, the landmark analyses avoided the inclusion of post-recovery samples and allowed for a between-group comparison based on equal temporal windows.

### 2.6. Contextual Variables

**Figure 4:**
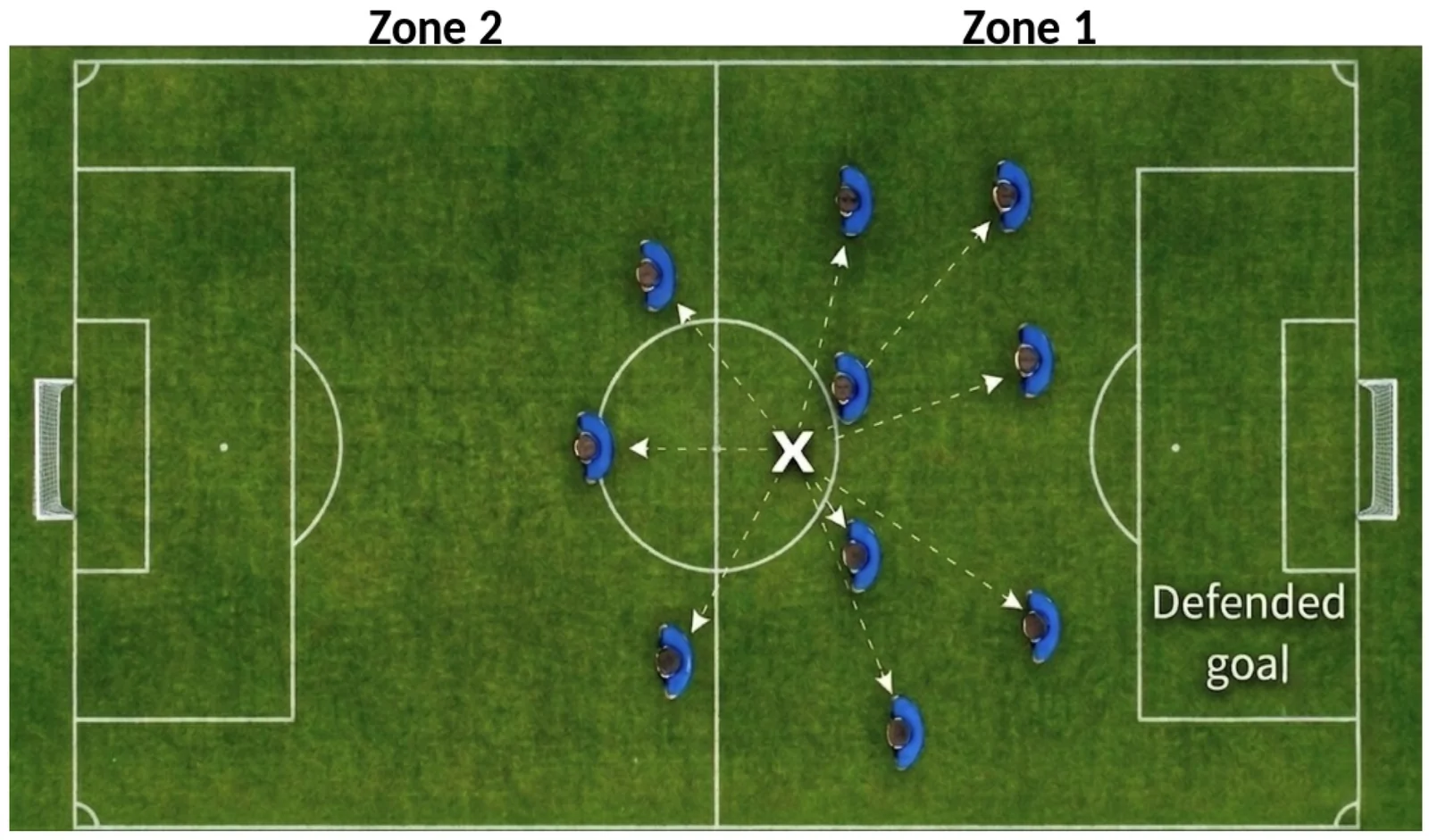
Schematic representation of the centroid-based zone classification. Episodes were assigned to Zone 1 or Zone 2 according to the predominant longitudinal position of the team centroid during non-possession.

The field zone of the episode was coded using the centroid of the analysed team (the team’s geometric centre) during the out-of-possession phase, operationally defined as the mean longitudinal position of the outfield players. The pitch was divided longitudinally with respect to the halfway line. Episodes in which the centroid was predominantly located in the defensive half were assigned to the middle defensive zone (Zone 1), whereas episodes in which the centroid was predominantly located in the offensive half were assigned to the middle offensive zone (Zone 2) (Figure 4; [22]). Each episode was assigned to the zone in which the centroid spent the greatest percentage of time [16]. Episodes were distributed between Zone 1 (*n* = 129) and Zone 2 (*n* = 119), with time spent in the prevalent zone always ≥84%, supporting the stability of the spatial classification. This classification captures only the longitudinal position of the centroid and does not represent lateral or depth information.

The following contextual variables were also coded: score state, home/away condition and match minute. These variables were subsequently included in the adjusted models because they may be associated with the team’s spatial organisation, tactical behaviour and speed of possession recovery, in line with previous studies on contextual variables in football [24]. Potential sources of bias were reduced through predefined criteria, blinded video verification and sensitivity analyses; however, contextual bias related to team strategy, opponents and match specificity may remain.

### 2.7. Statistical Analysis

#### 2.7.1. Analytical Hierarchy

**Figure 5:**
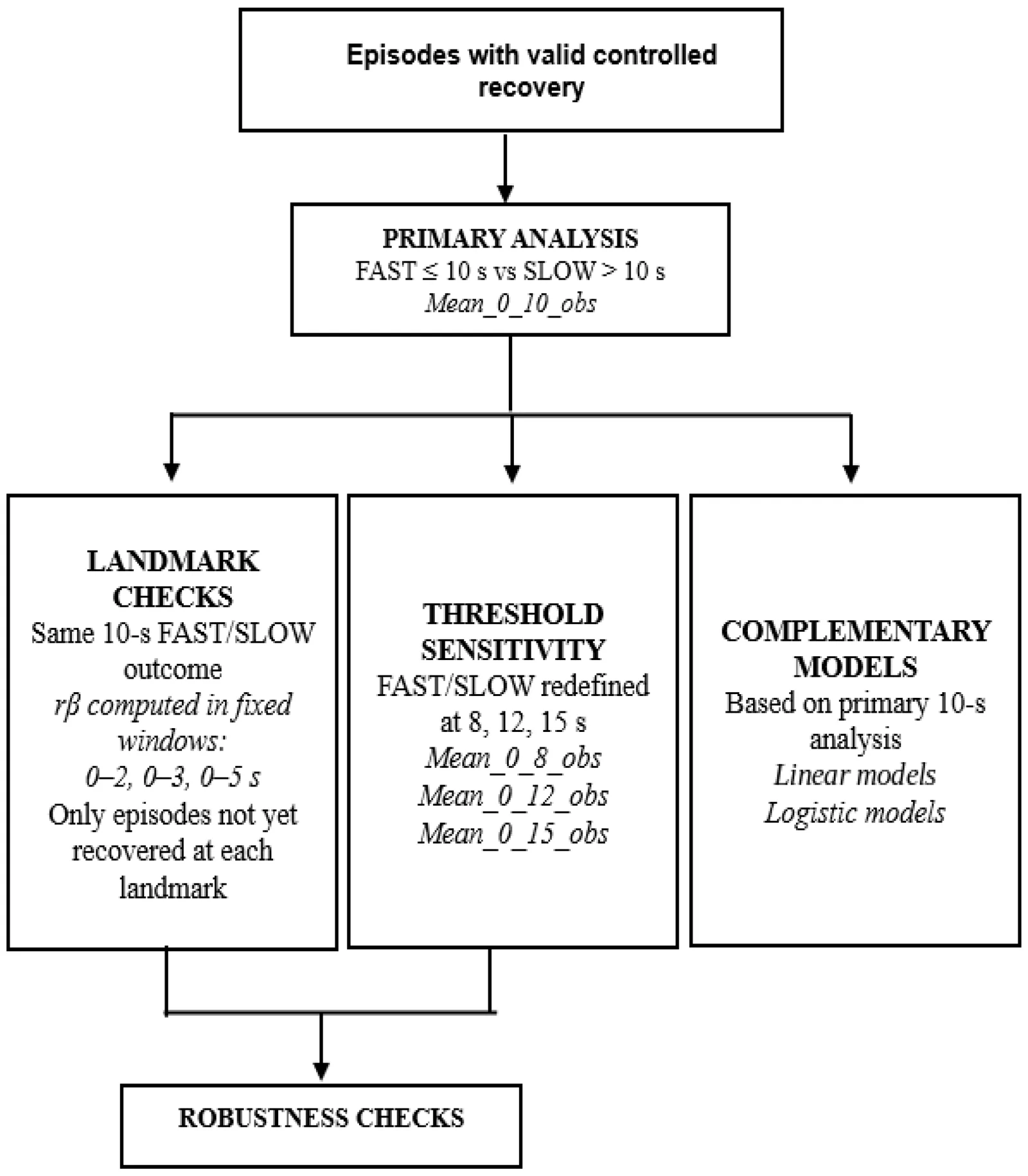
Predefined analytical hierarchy. The primary 10 s observed-window comparison was followed by temporal-threshold, fixed-window landmark, zone-stratified and technical sensitivity analyses and by complementary linear and logistic models.

Statistical analyses followed a predefined analytical hierarchy (Figure 5). The primary analysis compared Mean_0_10_obs between FAST and SLOW episodes using the 10 s threshold. Secondary analyses assessed the robustness of the result with respect to alternative temporal thresholds of 8, 12 and 15 s, landmark fixed-window analyses of 2, 3 and 5 s, and stratification by zone. Linear models, with match as a fixed effect and with a random intercept for match, and logistic regressions were used as complementary analyses to account, respectively, for the clustering of episodes by match and the association between rβ and FAST classification. Statistical analyses were performed in Python 3.13 (Python Software Foundation, Beaverton, OR, USA). No data were missing for any variable: complete observations were available for the rβ metric, the FAST/SLOW classification, the field zone and all contextual covariates across the 248 episodes. Consequently, no imputation or case-exclusion procedures were applied.

#### 2.7.2. Primary Comparison

Mean_0_10_obs was summarised using descriptive statistics for the whole sample and separately for FAST and SLOW episodes. The primary FAST–SLOW comparison was based on a two-tailed Welch’s *t*-test, because the main estimand was the mean between-group difference and this test does not assume equal variances between groups. The mean difference, 95% confidence interval, *p*-value and Cohen’s *d* were reported. As a robustness check, the Mann–Whitney *U* test was also used to evaluate whether the between-group pattern was consistent under a rank-based non-parametric approach. Confidence intervals for the between-group difference were also estimated using bootstrap with 5000 resamples. Cohen’s *d* was interpreted according to conventional thresholds: 0.20 = small, 0.50 = medium, and 0.80 = large [29]. As a descriptive check of stability across matches, the FAST–SLOW mean difference in the primary metric was calculated separately for each match.

#### 2.7.3. Sensitivity Comparisons

Sensitivity analyses based on alternative temporal thresholds and landmark fixed-window analyses were performed using the same statistical procedure as the primary between-group comparison. For each sensitivity metric, the FAST–SLOW comparison was assessed using a two-tailed Welch’s *t*-test, reporting the mean between-group difference, 95% confidence interval, *p*-value and Cohen’s *d*.

#### 2.7.4. Complementary Linear and Logistic Models

The clustering of episodes within matches was addressed in two complementary ways: by including match as a fixed effect and by estimating linear mixed-effects models with a random intercept for match. These models examined whether Mean_0_10_obs differed between FAST and SLOW episodes after accounting for match-level non-independence. The unadjusted model included FAST/SLOW group as the only fixed effect:

Mean_0_10_obs ~ FAST + (1|Match)(2)

An adjusted model additionally included contextual covariates:

Mean_0_10_obs ~ FAST + Zone + ScoreState + HomeAway + MinuteMatch + (1|Match)(3)

where Zone represented the prevalent field zone of the episode, ScoreState the score-line context, HomeAway the match location condition, and MinuteMatch the match minute.

An exploratory interaction model was also estimated to examine whether the FAST–SLOW difference varied according to field zone:

Mean_0_10_obs ~ FAST × Zone + ScoreState + HomeAway + MinuteMatch + (1|Match) (4)

Equations (2)–(4) are written in random-intercept notation; the corresponding match-adjusted specifications replace the random intercept (1|Match) with match as a fixed effect. Fixed effects from these models were reported as regression coefficients, 95% confidence intervals and *p*-values. Given the limited number of matches included, the results of the models with a random intercept for match were interpreted with caution and considered complementary to the FAST–SLOW comparison.

To evaluate the association between rβ and the tendency of an episode to be classified as FAST, logistic regression models were estimated with FAST/SLOW as the binary dependent variable and zMean_0_10_obs as the main predictor. Z-score standardisation was used exclusively to express the odds ratios per 1 standard deviation increase in the primary metric.

Three logistic models were estimated: unadjusted (Equation (5)), match-adjusted with MatchID as a fixed factor (Equation (6)), and adjusted for contextual variables, including Zone, ScoreState, HomeAway and MinuteMatch (Equation (7)), respectively:

FAST ~ zMean_0_10_obs(5)

FAST ~ zMean_0_10_obs + C(MatchID)(6)

FAST ~ zMean_0_10_obs + Zone + ScoreState + HomeAway + MinuteMatch(7)

MatchID was not included in the contextual model to avoid redundancy with variables partially nested within match, particularly HomeAway. Results were reported as odds ratios, 95% confidence intervals, and *p*-values. As an additional check, a mixed-effects logistic model with a random intercept for match was also estimated:

FAST ~ zMean_0_10_obs + (1|Match)(8)

This model was interpreted as a sensitivity analysis and not as the main inferential specification. All models were evaluated in terms of convergence, numerical stability and plausibility of the estimated parameters; model assumptions were additionally evaluated through residual diagnostics (normality and homoscedasticity), reported in Supplementary File S3. In the supplementary mixed-effects logistic models, the variance of the random intercept for match was also considered. The supplementary logistic mixed model was fitted by Bayesian maximum a posteriori estimation (BinomialBayesMixedGLM); its odds ratio, CI and *p*-value are Wald-type approximations. The level of statistical significance was set at *p* < 0.05.

As an additional supplementary check, for each sensitivity metric, a simple logistic model was estimated with the FAST/SLOW classification corresponding to the specific analysis as the dependent variable and the standardised rβ metric as the predictor. These models were reported exclusively in Supplementary Table S2 and interpreted as simple sensitivity analyses, not as main inferential specifications.

## 3. Results

### 3.1. Main FAST–SLOW Comparison

**Figure 6:**
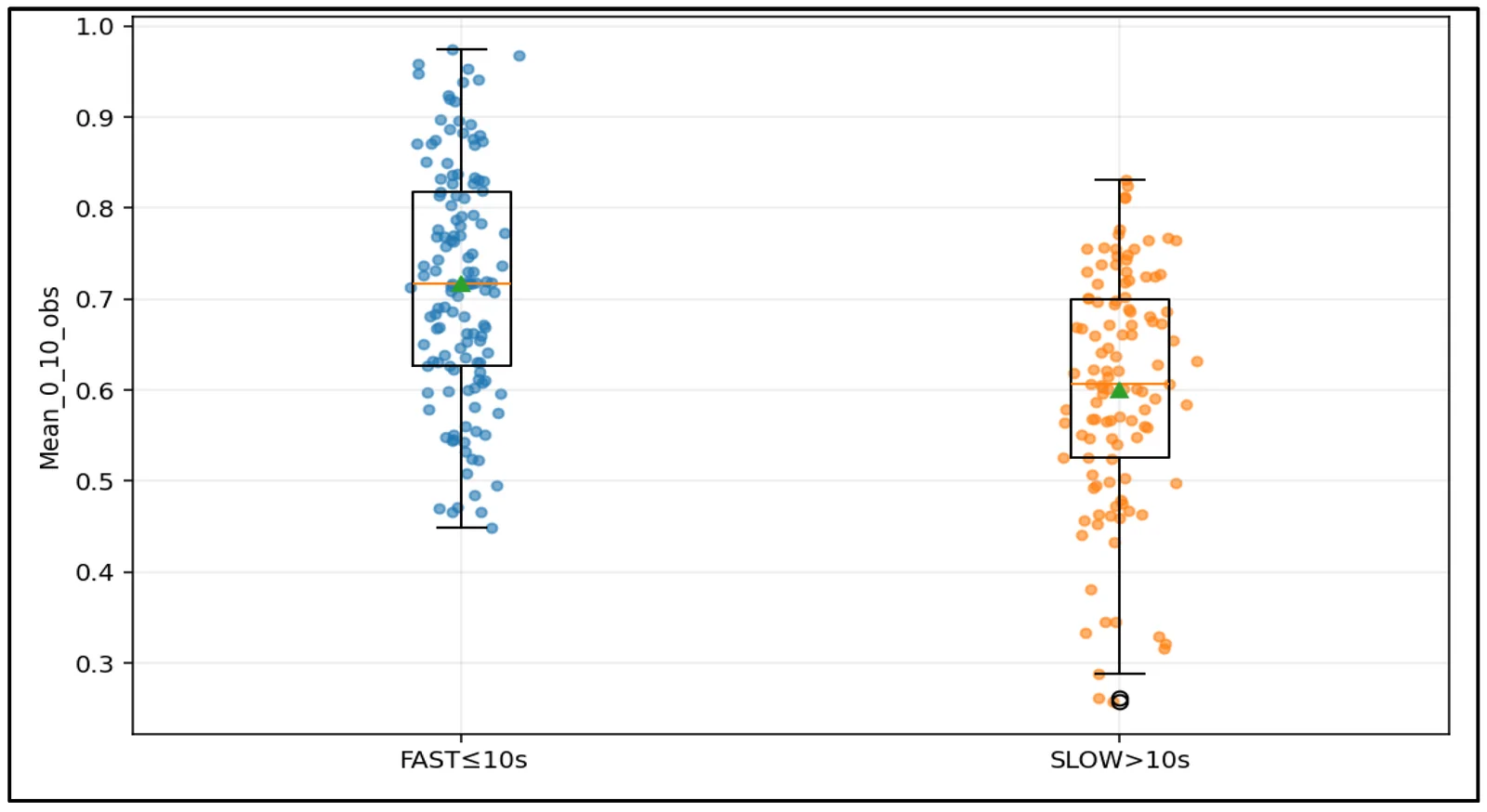
Distribution of Mean_0_10_obs in FAST and SLOW episodes. Points represent individual recovered negative-transition episodes; boxes and whiskers show median, interquartile range and standard boxplot spread; triangles indicate group means.

**Figure 7:**
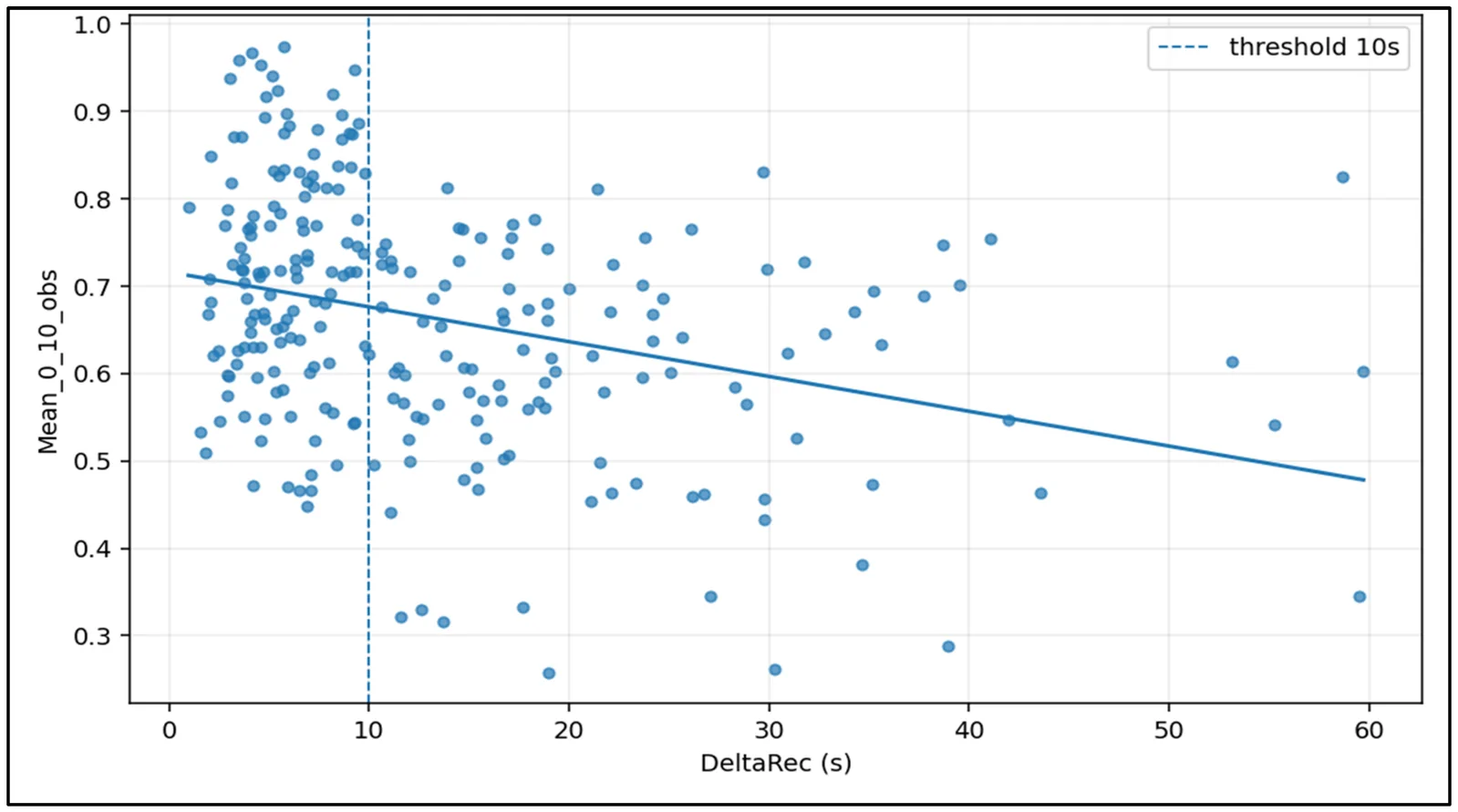
Relationship between recovery time and Mean_0_10_obs. Each point represents one recovered negative-transition episode; the dashed vertical line marks the 10 s FAST/SLOW threshold. The solid line is a simple linear regression fit: Mean_0_10_obs = 0.7162 − 0.0040 × DeltaRec_s (adjusted *R*^2^ = 0.097; *t*(246) = −5.25; *p* < 0.001).

FAST episodes showed higher mean values of Mean_0_10_obs than SLOW episodes (FAST: *M* = 0.717; SLOW: *M* = 0.600; difference = 0.117), with a statistically significant difference and a large effect size (Cohen’s *d* = 0.906, bootstrap 95% CI [0.67, 1.16]; *p* < 0.001). The full results of the main comparison and the zone-stratified analyses are reported in Table 1. The supporting non-parametric analysis was consistent with the main comparison (Mann–Whitney, *p* < 0.001, and *r* = 0.390; full output in Supplementary File S3). The mean resultant direction $\bar{\psi \beta }$ indicated that FAST episodes were collectively oriented toward the defended goal (circular mean = −4.8°; *R* = 0.45; Rayleigh *p* < 0.001; 78.2% of episodes within ±90° of the defended goal), whereas SLOW episodes showed no common orientation (−36.9°; *R* = 0.08; Rayleigh *p* = 0.48; 51.3%); the two groups differed significantly both in the absolute angular deviation from the direction to the defended goal (Mann–Whitney *U* test, *p* < 0.001) and in the proportion of episodes oriented within ±90° of that goal (χ^2^(1) = 18.6, *p* < 0.001). The distribution of individual values and the relationship between possession recovery time and Mean_0_10_obs are shown in Figure 6 and Figure 7, respectively. A simple linear regression confirmed a significant negative association between recovery time and Mean_0_10_obs (adjusted *R*^2^ = 0.097; *p* < 0.001; Figure 7). As a descriptive check of pattern stability across matches, the FAST–SLOW mean difference in the primary metric was positive in all five analysed matches (Match 1 = 0.125; Match 2 = 0.208; Match 3 = 0.103; Match 4 = 0.099; and Match 5 = 0.105), suggesting that the FAST > SLOW pattern was not driven by a single match.

As a technical check of the primary metric, the FAST > SLOW pattern remained directionally consistent across the alternative procedures for handling low-speed samples; a summary of these checks is reported in Supplementary Table S1. Zone-stratified analyses showed significant differences between FAST and SLOW episodes in both Zone 1 and Zone 2 (Zone 1: difference = 0.116; Zone 2: difference = 0.119; both *p* < 0.001).

### 3.2. Sensitivity Analyses

Sensitivity analyses confirmed the robustness of the FAST > SLOW pattern both with respect to the choice of temporal threshold and to the rβ calculation window. Using alternative thresholds of 8, 12 and 15 s, the differences between FAST and SLOW episodes remained positive and statistically significant, with effect sizes ranging from moderate to large. The same pattern was confirmed by the landmark fixed-window analyses in the first 2 s, 3 s and 5 s after t_l_, including only episodes that had not yet been resolved by the end of the window considered. The full results of the sensitivity analyses, including the effective samples of each comparison, are reported in Table 2. The supplementary simple logistic models for each sensitivity metric are reported in Supplementary Table S2.

### 3.3. Linear and Logistic Regression Models

In the model adjusted for match as a fixed effect, the effect of FAST was positive and statistically significant (estimate = 0.125; *p* < 0.001), and it remained significant when contextual covariates were added (estimate = 0.134; *p* < 0.001). The main results of the complementary linear and logistic models are summarised in Table 3, whereas the full model output is reported in Supplementary File S3. An exploratory interaction model with match as a fixed effect was also estimated. In this model, the effect of FAST at the reference level remained positive and statistically significant (estimate = 0.125; 95% CI [0.083, 0.167]; *p* < 0.001), whereas the FAST × zone interaction term was not significant (*p* = 0.530). In the corresponding linear mixed model with a random intercept for match, the estimate was 0.120 (95% CI [0.087, 0.153]; *p* < 0.001), with a random-intercept variance of 2.7 × 10^−6^ (ICC < 0.001): negligible, but not exactly zero. Consistent with this, the random-intercept estimate was close to the raw between-group difference (0.117), whereas the match-adjusted specification recovered the within-match difference (0.125).

**Figure 8:**
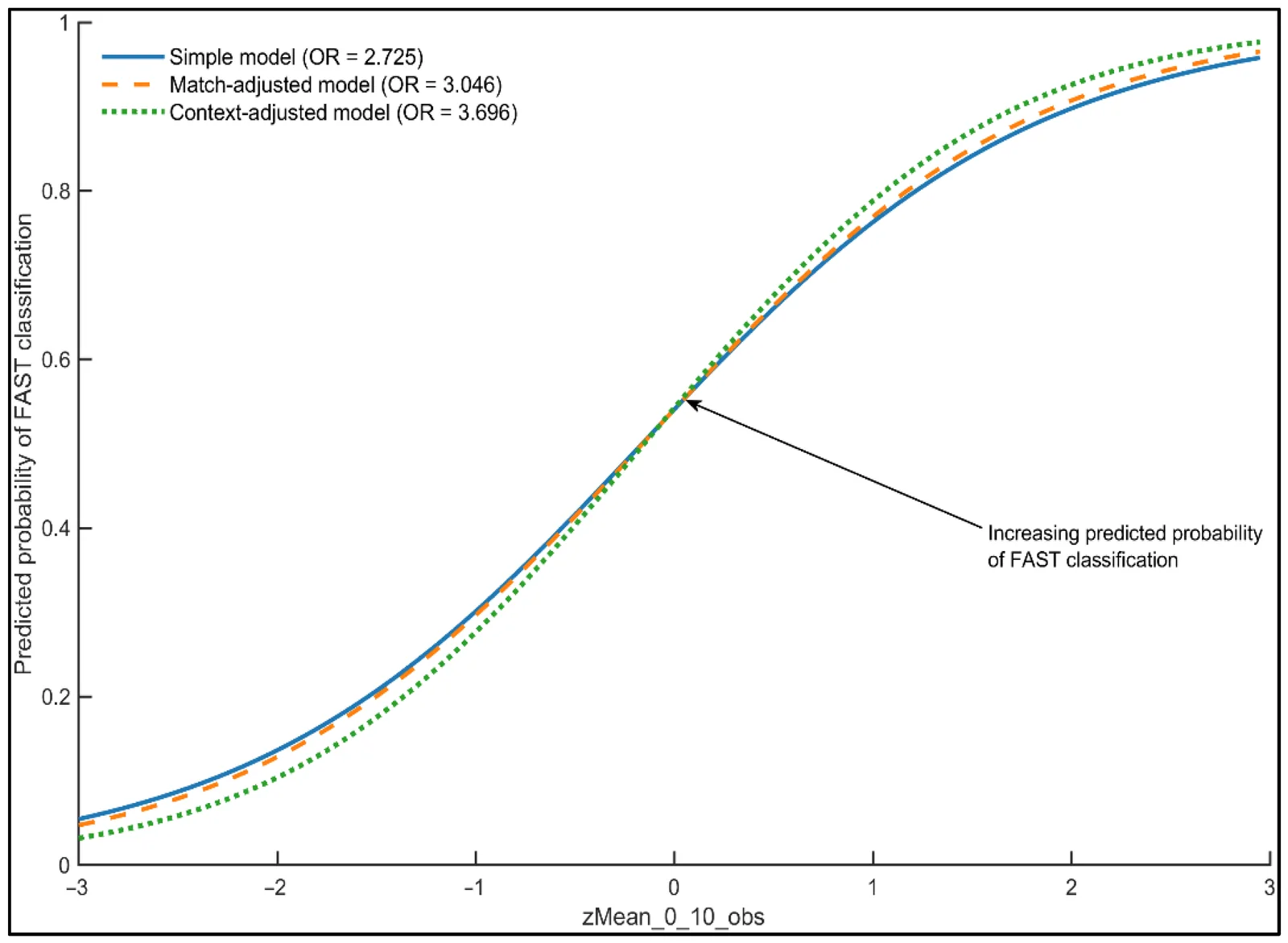
Predicted probability of FAST classification as a function of z-standardised Mean_0_10_obs. Curves represent the simple, match-adjusted and context-adjusted logistic models.

In the logistic regression models, higher zMean_0_10_obs values were associated with greater odds that an episode with valid recovery was classified as FAST rather than SLOW. The association was significant in the simple model, in the match-adjusted model and in the contextual model adjusted for zone, score state, home/away condition and match minute (OR = 2.725–3.696; all *p* < 0.001). The odds ratios and their confidence intervals are reported in Table 3, and the corresponding model-based predicted probability curves are shown in Figure 8.

As a supplementary check, the mixed-effects logistic model with a random intercept for match confirmed the same direction of the association between zMean_0_10_obs and FAST/SLOW classification (OR = 2.820; 95% CI [1.998, 3.979]; *p* < 0.001). The variance of the random intercept for match was 0.138, whereas in the linear mixed model it was negligible (2.7 × 10^−6^); the two random-effects specifications are therefore not directly comparable.

## 4. Discussion

The present study introduced rβ, a goal-referenced performance indicator representing the clustering of the players’ heading–goal angular deviations during the defensive phase of the match. rβ was examined as a process indicator of collective reorganisation after negative transitions (i.e., the time interval between ball loss and ball recovery). Our main hypothesis was that higher values of rβ during negative transitions would be associated with faster controlled possession recovery. This was confirmed by our main result, which showed that, among episodes ending with valid controlled recovery, greater collective angular concentration is associated with FAST classification, that is, possession recovery within 10 s. The contribution of the study therefore consists of proposing a task-anchored metric, derived from GNSS data, capable of linking the collective dynamics of the team to a practically relevant defensive outcome.

From an ecological dynamics and coordination dynamics perspective, loss of possession can be interpreted as a perturbation of the team system that requires rapid collective reorganisation, emerging from the interactions between teammates, opponents, the ball and space under new informational, spatial and temporal constraints [2,4,30]. Within this framework, rapid possession recovery does not depend exclusively on the action of the player closest to the ball but on the team’s collective ability to adapt in a coordinated manner to the new defensive problem. This process can be interpreted as a form of situated team decision-making, in which players continuously regulate their behaviour on the basis of the information available in the team–environment system which they perceive as shared affordances [11,31,32].

In this study, rβ should be interpreted as a team performance indicator capable of capturing a specific property of collective coordination: the concentration, or lower dispersion, of the error angles β of outfield players relative to the goal to be protected. Operationally, rβ recalls the logic of dimensional compression, as it synthesises multiple individual pieces of information into a single collective metric [10,33]. Higher values indicate greater collective coherence of the movement–goal relationship (coordinated movement, not its direction, which is given by $\bar{\psi \beta }$), which means that, as the attacking team recovered the ball, the players in the defending team are able to quickly coordinate their movements to block spaces that would allow for attackers to progress towards goal. As our results showed, this rapid reorganisation led the defensive team to be more effective in quickly regaining ball possession. However, the indicator does not directly measure the tactical precision of the response, nor does it imply that such coherence is always beneficial. Its functional relevance derives from the association observed, in the present sample, with rapid possession recovery.

The results fit within the literature using angular variables and collective variables to analyse properties of team coordination in football. Compared with classical spatial metrics, rβ introduces an explicit ecological reference, the goal to be protected, and quantifies the collective coherence of movement directions relative to a central objective of the defensive task: to block the progression of the attacking team [13,16]. In continuity with López-Felip et al. [16] and Carrilho et al. [12], who showed the utility of task-referenced angular variables and Kuramoto’s order parameter for analysing team coordination in football, the present study extends this analysis to negative transitions, adapting the angular reference to the goal to be protected.

Consistent with the definition of eco-physical variables as metrics oriented towards preserving goal-directedness and context-dependency, rβ can be interpreted as a possible eco-physical variable, because it maintains the link between players’ perceptual-motor behaviour, environmental reference and task goal [18].

This evidence aligns with the literature on defensive reaction time and counterpressing, in which recovery time describes the process outcome of the transition but does not describe how the team reorganises collectively in the seconds following possession loss [19,20,34]. In this sense, rβ helps link temporal outcome of recovery to a collective directional property observed during the post-loss window. Our findings also suggest that rapid ball recovery may not depend only on the immediate reaction after possession loss but also on the team’s collective organisation before and during the transition. If higher values of team coherence are associated with faster recoveries, then players may need to maintain a balanced collective structure while attacking, even when the primary objective is to progress and create scoring opportunities. Such anticipatory positioning may facilitate quicker reorganisation towards the team’s own goal immediately after possession loss. This interpretation supports the view that football performance should not be understood as a sequence of discrete phases but as a continuous process of collective coordination oriented towards emerging shared affordances. The sensitivity analyses strengthen the interpretation of rβ as a process indicator of immediate defensive reorganisation. The FAST > SLOW pattern remained stable with alternative temporal thresholds of 8, 12 and 15 s, suggesting that the association does not depend exclusively on the operational 10 s cut-off but reflects a more general relationship between collective directional coherence and recovery speed along the temporal continuum of negative transitions. Moreover, the landmark fixed-window analyses in the first 2, 3 and 5 s after $t_{l}$ showed the same pattern even when rβ was calculated over identical temporal windows between groups. This result reduces the ambiguity related to the different duration of the observed windows in FAST and SLOW episodes and suggests that the information contained in rβ is already present in the early phases of the transition, before episode resolution. Computed over identical intervals, these fixed-window analyses (Table 2) therefore provide the strongest evidence on this point. Because this difference was already present in a strictly pre-recovery window, reverse causation (convergence arising merely as a consequence of imminent recovery) is an unlikely sole explanation, although the direction of the association cannot be established from observational data.

Technical and contextual checks also support the robustness of the primary results. The consistency of the pattern after alternative thresholds for low-speed samples, exclusion of samples below the threshold and weighting by instantaneous speed indicates that the association was unlikely to be driven by numerical instabilities in the estimation of the heading angle (Supplementary Table S1). Similarly, the FAST–SLOW differences emerged in both Zone 1 and Zone 2, with no evidence of a significant interaction between group and zone, indicating that the relationship between rβ and rapid recovery was not dependent on the prevalent centroid-derived field zone of the episode. The complementary linear models further confirmed that the FAST–SLOW difference in Mean_0_10_obs remained positive even when accounting for the structure of episodes within matches and the main available contextual variables. A particularly relevant element derives from the logistic regressions: for each 1-SD increase in Mean_0_10_obs, the odds of FAST classification increased consistently in the unadjusted, match-adjusted and context-adjusted models, as illustrated in Figure 8. This result strengthens the interpretation of rβ as a process indicator capable of retaining discriminative information at the single-episode level and not only as an aggregated mean difference between FAST and SLOW episodes. Taken together, these concordant analyses constitute non-independent robustness checks on the same 248 episodes, not independent confirmations.

However, these results do not imply causality, nor do they indicate that high rβ values are necessarily beneficial. Rather, they show that, in the analysed sample, lower dispersion of the error angles relative to the goal to be protected was associated with faster resolution of the negative transition through controlled possession recovery.

Moreover, rβ cannot explain the outcome of a defensive transition on its own. The tactical meaning of the index remains context-dependent: similar values may reflect different processes if the team defends close to its own goal, counterpresses high or slows the opponent’s progression. This caution is consistent with evidence showing that collective behaviour in football is influenced by contextual and situational constraints [13,35]. In the present sample this ambiguity did not materialise: fast recoveries were directed toward the defended goal rather than into a high press, so their elevated rβ reflected coordinated recovery toward it. The mechanism linking this coherent retreat to faster recovery warrants further investigation.

### 4.1. Practical Applications

From an applied perspective, rβ can help practitioners prioritise which negative-transition episodes deserve closer video review: low post-transition values flag sequences in which the reaction appears fragmented, whereas high values point to a well-oriented collective response. It can also be used to profile a team’s transition behaviour and to contrast faster and slower recoveries across matches. rβ should be read together with technical–tactical analysis, considering pressure on the ball carrier, ball position, coverage of central spaces, opponent structure and protection of depth. In the present study, rβ was examined in relation to the speed of recovery, not to whether possession was regained.

Beyond the single match, rβ lends itself to longitudinal team monitoring and to supporting staff decisions. As it is obtained from the positional streams clubs already record, it can be followed throughout a season to observe how the defensive organisation shifts—for example, after a block of tactical work, a change in playing formation, or a period of fixture congestion—giving coaches an objective counterpart to their own reading of the game. Within the training process, and following the principle that practice tasks should represent the informational demands of competition [7,36], rβ could be computed during small-sided and large-sided games and compared with the values recorded in official matches, helping staff judge whether a drill reproduces the collective directional response of real negative transitions [37] and adjust its constraints accordingly; the same rationale offers a way to return targeted feedback to the players or units that most weaken the response in a given situation. rβ can also be combined with the physical-load metrics practitioners already compute [38] into a more complete, athlete-centred profile or fed into the centralised databases and dashboards on which analytical workflows increasingly depend [39,40,41]. These directions are in line with the use of tracking data for performance monitoring and decision-making in professional football [42,43,44].

Looking further ahead, computing rβ in real time would add a tactical layer to the in-game monitoring already carried out with GPS/GNSS systems, while extending it to other phases of play and to functional sub-units—defensive line, midfield and attack—would widen its applied reach. These uses, together with the player-level feedback and training-monitoring applications outlined above, are proposed as future possibilities and have not yet been empirically validated.

### 4.2. Limitations and Future Directions

The study has some limitations. First, only five official matches from a single professional team were analysed. This choice increases the internal coherence of the analysis but limits the generalisability of the results to other teams which might have different defensive principles, competitive levels, playing systems and pressing strategies. Because the 248 episodes are clustered within these five matches (a clustering for which the random-intercept models provided virtually no adjustment, given the negligible between-match variance), the inferential statistics should be read with caution, alongside the effect sizes, confidence intervals and match-level consistency of the effect.

Second, the observational design does not allow for causal inferences. Therefore, the association between rβ and rapid recovery should be interpreted as a tendency, not as evidence that greater directional coherence directly determines the outcome of the transition. Moreover, Mean_0_10_obs may be influenced by the different observed duration of FAST episodes, since the window stops at the moment of recovery. The 2, 3 and 5 s landmark fixed-window analyses controlled for this aspect through identical temporal windows between groups but should be interpreted as conservative checks on episodes still unresolved at the end of each window. The large dichotomised difference should therefore be read alongside the more modest continuous association, as dichotomising the outcome near its median can inflate the apparent effect size. Unmeasured confounders, such as opponent quality, pressing strategy and tactical style, may also have influenced the association.

Third, rβ does not directly include the position of the ball and opponents. Consequently, greater directional coherence relative to the goal to be protected should not automatically be interpreted as tactically optimal, since game affordances also depend on the spatio-temporal configuration of the ball, teammates and opponents [23]. Moreover, as only recovered transitions were analysed, rβ discriminates fast from slow recoveries, not recovery from non-recovery. Finally, future studies should verify the generalisability of the team performance indicator across multiple teams, playing systems and competitive levels, include transitions ending without controlled recovery, and calculate rβ at the level of functional subgroups, such as the defensive line, midfield and attack. Comparison between official matches and negative-transition drills could also clarify the effect of specific manipulations of task constraints.

## 5. Conclusions

The present study suggests that rβ, a collective measure of players’ directional coherence relative to the goal to be protected, is associated, among recovered transitions, with the speed of controlled possession recovery after negative transitions in professional football. Episodes with recovery within 10 s showed higher rβ values than episodes with later recovery, with a pattern supported by sensitivity analyses, landmark fixed-window analyses and complementary statistical models. The main contribution of the study consists of linking possession recovery time to a collective property of defensive reorganisation observed in the post-loss window, proposing a task-specific defensive application of a goal-referenced angular variable summarised using Kuramoto’s order parameter. rβ does not exhaust the complexity of defensive synergy nor directly measure the tactical correctness of the response, but it can be interpreted as a process indicator ecologically situating the task and supporting practitioners’ analysis and monitoring of collective defensive behaviour in professional football.

## Figures and Tables

**Table 1:** Primary comparison and zone-stratified analyses of Mean_0_10_obs. The primary comparison used the predefined 10 s FAST/SLOW threshold. Zone analyses were based on the prevalent centroid-derived field zone. FAST and SLOW values are group means, with the number of episodes reported in parentheses. Mean differences are expressed as FAST − SLOW. Effect sizes are reported as Cohen’s *d*.

Analysis	FAST, *M* (*n*)	SLOW, *M* (*n*)	Mean Difference	95% CI	Bootstrap 95% CI	*p*-Value	Effect Size
Primary comparison 10 s	0.717 (133)	0.600 (115)	0.117	[0.085, 0.150]	[0.086, 0.150]	<0.001	*d* = 0.906
Zone 1	0.717 (60)	0.601 (69)	0.116	[0.072, 0.160]	[0.074, 0.160]	<0.001	*d* = 0.926
Zone 2	0.718 (73)	0.598 (46)	0.119	[0.068, 0.171]	[0.070, 0.170]	<0.001	*d* = 0.877

**Table 2:** Sensitivity analyses based on alternative temporal thresholds and fixed-window landmarks. Threshold analyses reclassified FAST/SLOW episodes using 8, 12 and 15 s cut-offs and applied the same observed-window logic as the primary analysis. Landmark analyses retained the original 10 s FAST/SLOW classification and included only episodes still unresolved at the end of each fixed window. Values are group means, with the number of episodes reported in parentheses. Mean differences are expressed as FAST − SLOW.

Analysis	FAST, M (*n*)	SLOW, M (*n*)	Mean Difference	95% CI	*p*-Value	Effect Size
Sensitivity threshold 8 s	0.709 (107)	0.628 (141)	0.081	[0.047, 0.115]	<0.001	*d* = 0.599
Sensitivity threshold 12 s	0.706 (149)	0.598 (99)	0.108	[0.074, 0.142]	<0.001	*d* = 0.817
Sensitivity threshold 15 s	0.695 (167)	0.597 (81)	0.098	[0.063, 0.134]	<0.001	*d* = 0.734
Landmark 0–2 s	0.678 (128)	0.564 (115)	0.114	[0.066, 0.162]	<0.001	*d* = 0.614
Landmark 0–3 s	0.684 (118)	0.569 (115)	0.115	[0.071, 0.159]	<0.001	*d* = 0.681
Landmark 0–5 s	0.694 (80)	0.587 (115)	0.107	[0.063, 0.151]	<0.001	*d* = 0.674

**Table 3:** Summary of complementary linear and logistic models. Linear models report unstandardised estimates for FAST, whereas logistic models report odds ratios for a one-unit increase in zMean_0_10_obs, equivalent to a 1-SD increase in Mean_0_10_obs. Context-adjusted models included Zone, ScoreState, HomeAway and MinuteMatch. Only the primary terms of interest are shown.

Model	Term	Estimate/OR	95% CI	*p*-Value
Linear model, match-adjusted (match as fixed effect)	FAST	Estimate = 0.125	[0.093, 0.158]	<0.001
Linear model, match- and context-adjusted (match as fixed effect)	FAST	Estimate = 0.134	[0.103, 0.165]	<0.001
Linear mixed model, random intercept for match	FAST	Estimate = 0.120	[0.087, 0.153]	<0.001
Logistic model	zMean_0_10_obs	OR = 2.725	[1.959, 3.792]	<0.001
Logistic model, match-adjusted	zMean_0_10_obs	OR = 3.046	[2.134, 4.349]	<0.001
Logistic model, context-adjusted	zMean_0_10_obs	OR = 3.696	[2.501, 5.462]	<0.001

## Data Availability

The data are available from the corresponding author upon reasonable request. Due to confidentiality agreements with the professional football club and the technology provider (K-Sport World S.R.L., Pesaro, Italy), the datasets cannot be made publicly available. The analysis code used to compute rβ and to perform the statistical analyses is provided as Supplementary Materials (Supplementary File S2).

## Supplementary Materials

The following supporting information can be downloaded at https://www.mdpi.com/article/10.3390/sports14090374/s1. Supplementary File S1: Supplementary methods S1: Low-speed handling for heading-angle estimation; Table S1: Summary of technical sensitivity analyses for low-speed handling; Supplementary Results S1: Low-speed handling sensitivity analyses; Table S2: Simple logistic models for sensitivity metrics; Supplementary File S2: Analysis code; Supplementary File S3: Full statistical output; Supplementary File S4: Completed STROBE checklist.
