# Supplementary material for: Team Synergies in Professional Football: A Goal-Referenced Performance Indicator for Rapid Ball Recovery in Negative Transitions
Source: Sports (Basel). 2026 Sep 1;14(9):374. doi: 10.3390/sports14090374 (PMC13611748; doi:10.3390/sports14090374)
Supplement: Supplementary file 1 [file sports-14-00374-s001.zip › Supplementary_File_S1.pdf]

Supplementary Material

Supplementary Methods S1. Low-speed handling for heading-angle estimation

Heading angle φ was derived from the instantaneous velocity vector obtained by numerical differentiation of the x and y positional coordinates. Because the direction of the velocity vector becomes less stable and less informative at very low movement speeds, the primary manuscript specification treated samples with speed <0.35 m/s as low-informative for heading-angle estimation. These samples were not interpreted as missing positional data. Instead, they were circularly interpolated between adjacent valid heading-angle values to preserve temporal continuity and avoid artificial discontinuities around −π and +π.

Circular interpolation was performed on angular data to account for the periodic nature of φ. Specifically, when a low-speed sample occurred between two valid heading-angle values, interpolation was conducted in the circular domain rather than on raw linear angle values. This procedure avoided artefacts that could arise when interpolating across the −π/+π boundary.

To assess whether the findings depended on the selected low-speed threshold, rβ was recomputed using three alternative velocity thresholds: 0.20, 0.50 and 0.70 m/s. In these analyses, samples below each threshold were treated as low-informative and circularly interpolated using the same procedure as in the primary manuscript specification.

Two additional technical procedures were also tested at the primary 0.35 m/s threshold. First, below-threshold samples were excluded from the instantaneous computation of rβ, so that rβ at each time point was calculated only from players with valid heading-angle estimates. Second, a speed-weighted version of rβ was computed, in which each player's contribution to rβ was weighted by instantaneous movement speed. These analyses were interpreted as technical sensitivity analyses and not as separate tests of the main study hypothesis.

For the speed-weighted computation, rβ was calculated as:

$$r\beta,w(t) = |\Sigma_i\, w_i(t)\, e^{j\beta i(t)} / \Sigma_i\, w_i(t)|$$

where $w_i(t)$ represents the instantaneous speed of player i at time t. This formulation down-weights player samples with very low movement speed, for which heading direction is less informative.

For the exclusion procedure, rβ was calculated using only player samples with speed above the selected threshold. Therefore, N could vary across time points in this technical sensitivity analysis.

Supplementary Table S1. Summary of technical sensitivity analyses for low-speed handling

Technical check	Velocity threshold	Low-speed handling	Low-speed player-samples, %	Summary of result
**Alternative threshold 1**	0.20 m/s	Circular interpolation	3.21	FAST > SLOW direction preserved; $p < 0.001$
**Alternative threshold 2**	0.50 m/s	Circular interpolation	5.38	FAST > SLOW direction preserved; $p < 0.001$
**Alternative threshold 3**	0.70 m/s	Circular interpolation	7.74	FAST > SLOW direction preserved; $p < 0.001$
**Alternative handling 1**	0.35 m/s	Exclusion below threshold	4.15	FAST > SLOW direction preserved; $p < 0.001$
**Alternative handling 2**	0.35 m/s	Speed-weighted rβ	4.15	FAST > SLOW direction preserved; $p < 0.001$

Note. The primary manuscript specification used a 0.35 m/s velocity threshold with circular interpolation, as described in the main Methods. This table summarises alternative velocity thresholds and alternative low-speed handling procedures used to assess technical robustness. FAST and SLOW classifications were based on the predefined 10 s threshold. The table reports the direction and statistical consistency of the technical checks without duplicating the primary Mean_0_10_obs values reported in the main manuscript.

Supplementary Results S1. Low-speed handling sensitivity analyses

Across all technical sensitivity analyses, the FAST > SLOW pattern remained directionally consistent with the primary analysis. Recomputing rβ using alternative low-speed thresholds, excluding below-threshold samples, or applying speed-weighted rβ did not change the direction of the group difference. All alternative technical sensitivity analyses remained statistically significant. These findings suggest that the association between observed-window rβ and FAST/SLOW classification was unlikely to be driven by the specific low-speed handling procedure used for heading-angle estimation.

Supplementary Table S2. Simple logistic models for sensitivity metrics

Analysis	Logistic model	Predictor	OR	95% CI	*p*-value
**Threshold 8 s**	FAST8 ~ zMean_0_8_obs	zMean_0_8_obs	1.95	[1.46, 2.61]	< 0.001
**Threshold 12 s**	FAST12 ~ zMean_0_12_obs	zMean_0_12_obs	2.31	[1.69, 3.16]	< 0.001
**Threshold 15 s**	FAST15 ~ zMean_0_15_obs	zMean_0_15_obs	1.95	[1.45, 2.63]	< 0.001
**Landmark 0–2 s**	FAST_landmark2 ~ zMean_0_2_landmark	zMean_0_2_landmark	1.88	[1.42, 2.49]	< 0.001
**Landmark 0–3 s**	FAST_landmark3 ~ zMean_0_3_landmark	zMean_0_3_landmark	2.04	[1.52, 2.74]	< 0.001
**Landmark 0–5 s**	FAST_landmark5 ~ zMean_0_5_landmark	zMean_0_5_landmark	2.04	[1.46, 2.83]	< 0.001

Note. Logistic models were fitted separately for each sensitivity metric. Threshold models used the FAST/SLOW classification and observed-window rβ metric corresponding to each temporal threshold. Landmark models retained the original 10 s FAST/SLOW logic and used the corresponding fixed-window rβ metric as standardised predictor. OR values refer to a one-unit increase in the standardised predictor.
