# Supplementary material for: Team Synergies in Professional Football: A Goal-Referenced Performance Indicator for Rapid Ball Recovery in Negative Transitions
Source: Sports (Basel). 2026 Sep 1;14(9):374. doi: 10.3390/sports14090374 (PMC13611748; doi:10.3390/sports14090374)
Supplement: Supplementary file 1 [file sports-14-00374-s001.zip › Supplementary_File_S2_Analysis_code.pdf]

```python
"""
Statistical analysis code for:
Team synergies in professional football: a goal-referenced performance indicator
for rapid ball recovery in negative transitions

Software: Python 3.13

This script contains only the analytical code needed to document the
calculations reported in the manuscript.

Raw tracking data, video data, and the final analysis-ready dataset are not
distributed with this code release because of club and provider restrictions.
Accordingly, the code is provided as a clean reference implementation of the
computational and statistical procedures described in the paper.

Expected episode-level variables if data are available
------------------------------------------------------
- Primary dataset:
- Episode_ID
- MatchID
- DeltaRec_s
- FastSlow
- Mean_0_10_obs
- Zone
- ScoreState
- HomeAway
- MinuteMatch

Threshold sensitivity metrics:
- FastSlow_8s
- Mean_0_8_obs
- FastSlow_12s
- Mean_0_12_obs
- FastSlow_15s
- Mean_0_15_obs

Landmark metrics:
- Mean_0_2_fix
- Mean_0_3_fix
- Mean_0_5_fix

Technical low-speed metrics:
- Mean_0_10_lowspd_interp_020
- Mean_0_10_lowspd_interp_035
- Mean_0_10_lowspd_interp_050
- Mean_0_10_lowspd_interp_070
- Mean_0_10_lowspd_exclude_035
- Mean_0_10_lowspd_weighted_035

Technical low-speed sample proportions, if available:
- LowSpeedPct_interp_020
- LowSpeedPct_interp_035
- LowSpeedPct_interp_050
- LowSpeedPct_interp_070
- LowSpeedPct_exclude_035
- LowSpeedPct_weighted_035
"""

from __future__ import annotations

import math

import numpy as np
import pandas as pd
from scipy import stats
import statsmodels.formula.api as smf
from statsmodels.genmod.bayes_mixed_glm import BinomialBayesMixedGLM


N_BOOT = 5000
BOOT_SEED = 42
```

```python
# ---------------------------------------------------------------------------
# Angular variables and r_beta
# ---------------------------------------------------------------------------

def circular_difference(angle1: np.ndarray, angle2: np.ndarray) -> np.ndarray:
    """Circular difference in [-pi, +pi]."""
    return np.angle(np.exp(1j * (np.asarray(angle1) - np.asarray(angle2))))


def compute_goal_angle(x: np.ndarray, y: np.ndarray, goal_x: float, goal_y:
    float) -> np.ndarray:
    """Goal angle psi_g."""
    return np.arctan2(goal_y - np.asarray(y), goal_x - np.asarray(x))


def circular_interpolate(angle_series: np.ndarray, valid_mask: np.ndarray) -> np.ndarray:
    """Circular interpolation used for low-speed heading-angle handling."""
    angle_series = np.asarray(angle_series, dtype=float)
    valid_mask = np.asarray(valid_mask, dtype=bool)
    if valid_mask.sum() == 0:
        return np.full_like(angle_series, np.nan, dtype=float)
    idx = np.arange(angle_series.size, dtype=float)
    unwrapped = np.unwrap(angle_series[valid_mask])
    interpolated = np.interp(idx, idx[valid_mask], unwrapped)
    return np.angle(np.exp(1j * interpolated))


def compute_heading_angle(
    x: np.ndarray,
    y: np.ndarray,
    dt: float = 0.1,
    low_speed_threshold: float = 0.35,
    mode: str = "interpolate",
) -> tuple[np.ndarray, np.ndarray]:
    """
    Heading angle phi from numerical differentiation of x and y.

    mode:
    - "interpolate": circular interpolation below threshold
    - "exclude": below-threshold samples excluded
    """
    x = np.asarray(x, dtype=float)
    y = np.asarray(y, dtype=float)
    vx = np.gradient(x, dt)
    vy = np.gradient(y, dt)
    speed = np.sqrt(vx**2 + vy**2)
    phi = np.arctan2(vy, vx)
    valid = np.isfinite(phi) & np.isfinite(speed) & (speed >= float(low_speed_threshold))

    if mode == "interpolate":
        phi_out = circular_interpolate(phi, valid)
    elif mode == "exclude":
        phi_out = phi.copy()
        phi_out[~valid] = np.nan
    else:
        raise ValueError(f"Unsupported low-speed mode: {mode}")
    return phi_out, speed


def compute_r_beta(beta_values: np.ndarray) -> float:
    """Kuramoto order parameter."""
    beta_values = np.asarray(beta_values, dtype=float)
    beta_values = beta_values[np.isfinite(beta_values)]
    if beta_values.size == 0:
        return float("nan")
    return float(np.abs(np.mean(np.exp(1j * beta_values))))


def compute_speed_weighted_r_beta(beta_values: np.ndarray, speed_values: np.ndarray) -> float:
    """Speed-weighted r_beta used in the technical sensitivity analysis."""
    beta_values = np.asarray(beta_values, dtype=float)
```

```
    speed_values = np.asarray(speed_values, dtype=float)
    mask = np.isfinite(beta_values) & np.isfinite(speed_values) & (speed_values > 0)
    if mask.sum() == 0:
        return float("nan")
    num = np.sum(speed_values[mask] * np.exp(1j * beta_values[mask]))
    den = np.sum(speed_values[mask])
    return float(np.abs(num / den))


def compute_observed_window_mean(r_beta_series: np.ndarray, recovery_time_s: float,
    threshold_s: float = 10.0, dt: float = 0.1) -> float:
    """
    Observed-window mean:
    - FAST episodes: t0 to recovery
    - SLOW episodes: t0 to threshold
    """
    end_time = min(float(recovery_time_s), float(threshold_s))
    n_frames = max(1, int(round(end_time / dt)))
    values = np.asarray(r_beta_series, dtype=float)[:n_frames]
    values = values[np.isfinite(values)]
    if values.size == 0 or end_time <= 0:
        return float("nan")
    return float(np.sum(values) * dt / end_time)


def compute_fixed_window_mean(r_beta_series: np.ndarray, window_s: float, dt: float =
    0.1) -> float:
    """Fixed-window mean used for 0-2 s, 0-3 s and 0-5 s landmark analyses."""
    n_frames = max(1, int(round(float(window_s) / dt)))
    values = np.asarray(r_beta_series, dtype=float)[:n_frames]
    values = values[np.isfinite(values)]
    if values.size == 0:
        return float("nan")
    return float(np.mean(values))


# ---------------------------------------------------------------------------
# Statistical helpers
# ---------------------------------------------------------------------------

def zscore(series: pd.Series) -> pd.Series:
    sd = float(series.std(ddof=1))
    if not np.isfinite(sd) or sd <= 0:
        return pd.Series(np.zeros(len(series), dtype=float), index=series.index)
    return (series - float(series.mean())) / sd


def cohens_d(x: np.ndarray, y: np.ndarray) -> float:
    pooled_sd = math.sqrt(
        (((len(x) - 1) * np.var(x, ddof=1)) + ((len(y) - 1) * np.var(y, ddof=1)))
        / (len(x) + len(y) - 2)
    )
    return float((np.mean(x) - np.mean(y)) / pooled_sd)


def welch_ci_mean_diff(x: np.ndarray, y: np.ndarray, alpha: float = 0.05) -> tuple[float,
    float]:
    mx, my = float(np.mean(x)), float(np.mean(y))
    vx, vy = float(np.var(x, ddof=1)), float(np.var(y, ddof=1))
    se2 = (vx / len(x)) + (vy / len(y))
    se = math.sqrt(se2)
    df_num = se2**2
    df_den = ((vx / len(x)) ** 2) / (len(x) - 1) + ((vy / len(y)) ** 2) / (len(y) - 1)
    df = df_num / df_den
    tcrit = float(stats.t.ppf(1.0 - alpha / 2.0, df))
    diff = mx - my
    return diff - tcrit * se, diff + tcrit * se


def bootstrap_mean_difference(x: np.ndarray, y: np.ndarray, n_boot: int = N_BOOT, seed: int =
    BOOT_SEED) -> tuple[float, float]:
    rng = np.random.default_rng(seed)
```

```
    diffs = []
    for _ in range(n_boot):
        xb = rng.choice(x, size=len(x), replace=True)
        yb = rng.choice(y, size=len(y), replace=True)
        diffs.append(float(np.mean(xb) - np.mean(yb)))
    return tuple(np.percentile(diffs, [2.5, 97.5]))


def mann_whitney_r(x: np.ndarray, y: np.ndarray) -> tuple[float, float, float]:
    res = stats.mannwhitneyu(x, y, alternative="two-sided")
    n1, n2 = len(x), len(y)
    mean_u = n1 * n2 / 2.0
    sd_u = math.sqrt(n1 * n2 * (n1 + n2 + 1) / 12.0)
    z = (float(res.statistic) - mean_u) / sd_u
    r = abs(z) / math.sqrt(n1 + n2)
    return float(res.statistic), float(res.pvalue), float(r)


def compare_groups(df: pd.DataFrame, group_col: str, metric_col: str) -> dict:
    d = df[[group_col, metric_col]].dropna().copy()
    fast = pd.to_numeric(d.loc[d[group_col] == "FAST", metric_col],
        errors="coerce").dropna().to_numpy(dtype=float)
    slow = pd.to_numeric(d.loc[d[group_col] == "SLOW", metric_col],
        errors="coerce").dropna().to_numpy(dtype=float)

    t_res = stats.ttest_ind(fast, slow, equal_var=False)
    ci_low, ci_high = welch_ci_mean_diff(fast, slow)
    boot_low, boot_high = bootstrap_mean_difference(fast, slow)
    u_stat, p_mw, r_mw = mann_whitney_r(fast, slow)
    d_boot_low, d_boot_high = bootstrap_cohens_d(fast, slow)
    auc = auc_from_mann_whitney(fast, slow)

    return {
        "FAST_mean": float(np.mean(fast)),
        "FAST_n": int(len(fast)),
        "SLOW_mean": float(np.mean(slow)),
        "SLOW_n": int(len(slow)),
        "Difference": float(np.mean(fast) - np.mean(slow)),
        "CI95_low": ci_low,
        "CI95_high": ci_high,
        "Bootstrap_CI95_low": boot_low,
        "Bootstrap_CI95_high": boot_high,
        "Welch_t": float(t_res.statistic),
        "Welch_df": float(t_res.df),
        "Welch_p": float(t_res.pvalue),
        "Cohens_d": cohens_d(fast, slow),
        "Cohens_d_boot_CI95_low": d_boot_low,
        "Cohens_d_boot_CI95_high": d_boot_high,
        "MW_U": u_stat,
        "MW_p": p_mw,
        "MW_r": r_mw,
        "AUC": auc["AUC"],
        "AUC_CI95_low": auc["CI95_low"],
        "AUC_CI95_high": auc["CI95_high"],
    }


def logistic_or_ci(model, term: str) -> dict:
    beta = float(model.params[term])
    se = float(model.bse[term])
    return {
        "Estimate": beta,
        "SE": se,
        "OR": float(np.exp(beta)),
        "CI95_low": float(np.exp(beta - 1.96 * se)),
        "CI95_high": float(np.exp(beta + 1.96 * se)),
        "p_value": float(model.pvalues[term]),
    }


# ---------------------------------------------------------------------------
# Analyses reported in the manuscript
```

```python
# --------------------------------------------------------------------------


def sample_description(df: pd.DataFrame) -> pd.DataFrame:
    return pd.DataFrame(
        [
            {
                "Total_episodes": int(len(df)),
                "FAST_n": int((df["FastSlow"] == "FAST").sum()),
                "SLOW_n": int((df["FastSlow"] == "SLOW").sum()),
                "FAST_pct": float((df["FastSlow"] == "FAST").mean() * 100.0),
                "SLOW_pct": float((df["FastSlow"] == "SLOW").mean() * 100.0),
                "Mean_recovery_time_s": float(pd.to_numeric(df["DeltaRec_s"],
                    errors="coerce").mean()),
            }
        ]
    )


def primary_comparison(df: pd.DataFrame) -> dict:
    return compare_groups(df, "FastSlow", "Mean_0_10_obs")


def zone_stratified_analyses(df: pd.DataFrame) -> pd.DataFrame:
    rows = []
    for zone in sorted(pd.to_numeric(df["Zone"],
        errors="coerce").dropna().astype(int).unique().tolist()):
        dz = df[pd.to_numeric(df["Zone"], errors="coerce") == zone].copy()
        rows.append({"Zone": zone, **compare_groups(dz, "FastSlow", "Mean_0_10_obs")})
    return pd.DataFrame(rows)


def match_level_differences(df: pd.DataFrame) -> pd.DataFrame:
    rows = []
    for match_id in sorted(pd.to_numeric(df["MatchID"],
        errors="coerce").dropna().astype(int).unique().tolist()):
        dm = df[pd.to_numeric(df["MatchID"], errors="coerce") == match_id].copy()
        fast_mean = float(dm.loc[dm["FastSlow"] == "FAST", "Mean_0_10_obs"].mean())
        slow_mean = float(dm.loc[dm["FastSlow"] == "SLOW", "Mean_0_10_obs"].mean())
        rows.append({"MatchID": match_id, "FAST_minus_SLOW": fast_mean - slow_mean})
    return pd.DataFrame(rows)


def threshold_sensitivity_analyses(df: pd.DataFrame) -> pd.DataFrame:
    specs = [
        ("Sensitivity threshold 8 s", "FastSlow_8s", "Mean_0_8_obs"),
        ("Sensitivity threshold 12 s", "FastSlow_12s", "Mean_0_12_obs"),
        ("Sensitivity threshold 15 s", "FastSlow_15s", "Mean_0_15_obs"),
    ]
    rows = []
    for label, group_col, metric_col in specs:
        rows.append({"Analysis": label, **compare_groups(df, group_col, metric_col)})
    return pd.DataFrame(rows)


def landmark_fixed_window_analyses(df: pd.DataFrame) -> pd.DataFrame:
    specs = [
        ("Landmark 0-2 s", "Mean_0_2_fix", 2.0),
        ("Landmark 0-3 s", "Mean_0_3_fix", 3.0),
        ("Landmark 0-5 s", "Mean_0_5_fix", 5.0),
    ]
    rows = []
    for label, metric_col, window_s in specs:
        d = df[pd.to_numeric(df["DeltaRec_s"], errors="coerce") > window_s].copy()
        rows.append({"Analysis": label, **compare_groups(d, "FastSlow", metric_col)})
    return pd.DataFrame(rows)


def supplementary_table_s1_low_speed(df: pd.DataFrame) -> pd.DataFrame:
    specs = [
        ("Alternative threshold 1", 0.20, "Circular interpolation",
            "Mean_0_10_lowspd_interp_020", "LowSpeedPct_interp_020"),
        ("Alternative threshold 2", 0.50, "Circular interpolation",
```

```
        "Mean_0_10_lowspd_interp_050", "LowSpeedPct_interp_050"),
    ("Alternative threshold 3", 0.70, "Circular interpolation",
        "Mean_0_10_lowspd_interp_070", "LowSpeedPct_interp_070"),
    ("Alternative handling 1", 0.35, "Exclusion below threshold",
        "Mean_0_10_lowspd_exclude_035", "LowSpeedPct_exclude_035"),
    ("Alternative handling 2", 0.35, "Speed-weighted r_beta",
        "Mean_0_10_lowspd_weighted_035", "LowSpeedPct_weighted_035"),
]
rows = []
for label, threshold, handling, metric_col, pct_col in specs:
    cmp = compare_groups(df, "FastSlow", metric_col)
    low_speed_pct = float(pd.to_numeric(df[pct_col],
        errors="coerce").dropna().mean()) if pct_col in df.columns else float("nan")
    rows.append(
        {
            "Technical_check": label,
            "Velocity_threshold_mps": threshold,
            "Low_speed_handling": handling,
            "Low_speed_player_samples_pct": low_speed_pct,
            "Summary_of_result": (
                "FAST > SLOW direction preserved; p < 0.001"
                if cmp["Welch_p"] < 0.001 and cmp["Difference"] > 0
                else "Check result"
            ),
        }
    )
return pd.DataFrame(rows)


def supplementary_table_s2_logistic(df: pd.DataFrame) -> pd.DataFrame:
    rows = []

    threshold_specs = [
        ("Threshold 8 s", "FastSlow_8s", "Mean_0_8_obs", "zMean_0_8_obs"),
        ("Threshold 12 s", "FastSlow_12s", "Mean_0_12_obs", "zMean_0_12_obs"),
        ("Threshold 15 s", "FastSlow_15s", "Mean_0_15_obs", "zMean_0_15_obs"),
    ]
    for label, group_col, metric_col, predictor_label in threshold_specs:
        d = df[[group_col, metric_col]].dropna().copy()
        d["FAST_binary"] = np.where(d[group_col] == "FAST", 1, 0)
        d[predictor_label] = zscore(pd.to_numeric(d[metric_col], errors="coerce"))
        model = smf.logit(f"FAST_binary ~ {predictor_label}", data=d).fit(disp=False)
        rows.append({"Analysis": label, "Predictor": predictor_label, **logistic_or_ci(model,
            predictor_label)})

    landmark_specs = [
        ("Landmark 0-2 s", "Mean_0_2_fix", 2.0, "zMean_0_2_landmark"),
        ("Landmark 0-3 s", "Mean_0_3_fix", 3.0, "zMean_0_3_landmark"),
        ("Landmark 0-5 s", "Mean_0_5_fix", 5.0, "zMean_0_5_landmark"),
    ]
    for label, metric_col, window_s, predictor_label in landmark_specs:
        d = df[pd.to_numeric(df["DeltaRec_s"], errors="coerce") > window_s].copy()
        d = d[["FastSlow", metric_col]].dropna().copy()
        d["FAST_binary"] = np.where(d["FastSlow"] == "FAST", 1, 0)
        d[predictor_label] = zscore(pd.to_numeric(d[metric_col], errors="coerce"))
        model = smf.logit(f"FAST_binary ~ {predictor_label}", data=d).fit(disp=False)
        rows.append({"Analysis": label, "Predictor": predictor_label, **logistic_or_ci(model,
            predictor_label)})

    return pd.DataFrame(rows)


def linear_models_match_fixed(df: pd.DataFrame) -> pd.DataFrame:
    """
    Linear models with match entered as a FIXED effect.
    These are the specifications reported in Table 3 of the manuscript as
    "Linear model, match-adjusted (match as fixed effect)" and "Linear model,
    match- and context-adjusted (match as fixed effect)", together with the
    exploratory FAST x Zone interaction model described in Section 3.3.
    They estimate the WITHIN-match FAST-SLOW difference and are therefore
    complementary to the random-intercept specifications fitted in
    linear_mixed_models(), which instead return an estimate close to the raw
```

```python
    between-group difference when the between-match variance is negligible.
    Wald p-values use the same normal approximation adopted for the mixed
    models, so that the two families of estimates are directly comparable.
    """
    d = df.copy()
    d["FAST_binary"] = np.where(d["FastSlow"] == "FAST", 1.0, 0.0)
    specs = [
        ("Linear model, match-adjusted (match as fixed effect)",
         "Mean_0_10_obs ~ FAST_binary + C(MatchID)"),
        ("Linear model, match- and context-adjusted (match as fixed effect)",
         "Mean_0_10_obs ~ FAST_binary + C(Zone) + C(ScoreState) + C(HomeAway)"
         " + MinuteMatch + C(MatchID)"),
        ("Interaction linear model (match as fixed effect)",
         "Mean_0_10_obs ~ FAST_binary * C(Zone) + C(ScoreState) + C(HomeAway)"
         " + MinuteMatch + C(MatchID)"),
    ]
    rows = []
    for label, formula in specs:
        m = smf.ols(formula, data=d).fit()
        b = float(m.params["FAST_binary"])
        se = float(m.bse["FAST_binary"])
        row = {
            "Model": label,
            "Estimate": b,
            "SE": se,
            "CI95_low": b - 1.96 * se,
            "CI95_high": b + 1.96 * se,
            "Wald_z": b / se,
            "p_value": float(2.0 * (1.0 - stats.norm.cdf(abs(b / se)))),
            "Residual_variance": float(m.mse_resid),
        }
        if "*" in formula:
            zs = [abs(float(m.params[t]) / float(m.bse[t]))
                  for t in m.params.index if "FAST_binary:C(Zone)" in t]
            ps = [float(2.0 * (1.0 - stats.norm.cdf(z))) for z in zs]
            row["Interaction_min_p"] = float(np.min(ps)) if ps else float("nan")
        rows.append(row)
    return pd.DataFrame(rows)


def linear_mixed_models(df: pd.DataFrame) -> pd.DataFrame:
    d = df.copy()
    d["FAST_binary"] = np.where(d["FastSlow"] == "FAST", 1.0, 0.0)

    rows = []

    m1 = smf.mixedlm("Mean_0_10_obs ~ FAST_binary", data=d,
        groups=d["MatchID"]).fit(reml=False, method="lbfgs", disp=False)
    b1 = float(m1.params["FAST_binary"])
    se1 = float(m1.bse["FAST_binary"])
    rows.append(
        {
            "Model": "Linear mixed model, random intercept for match",
            "Estimate": b1,
            "SE": se1,
            "CI95_low": b1 - 1.96 * se1,
            "CI95_high": b1 + 1.96 * se1,
            "p_value": float(2.0 * (1.0 - stats.norm.cdf(abs(b1 / se1)))),
            "RandomInterceptVar_Match": float(m1.cov_re.iloc[0, 0]),
            "ICC_Match": (float(m1.cov_re.iloc[0, 0])
                          / (float(m1.cov_re.iloc[0, 0]) + float(m1.scale))),
        }
    )

    m2 = smf.mixedlm(
        "Mean_0_10_obs ~ FAST_binary + C(Zone) + C(ScoreState) + C(HomeAway) + MinuteMatch",
        data=d,
        groups=d["MatchID"],
    ).fit(reml=False, method="lbfgs", disp=False)
    b2 = float(m2.params["FAST_binary"])
    se2 = float(m2.bse["FAST_binary"])
    rows.append(
```

```python
        {
            "Model": "Linear mixed model, random intercept, context-adjusted",
            "Estimate": b2,
            "SE": se2,
            "CI95_low": b2 - 1.96 * se2,
            "CI95_high": b2 + 1.96 * se2,
            "p_value": float(2.0 * (1.0 - stats.norm.cdf(abs(b2 / se2)))),
        }
    )

    m3 = smf.mixedlm(
        "Mean_0_10_obs ~ FAST_binary * C(Zone) + C(ScoreState) + C(HomeAway) + MinuteMatch",
        data=d,
        groups=d["MatchID"],
    ).fit(reml=False, method="lbfgs", disp=False)
    b3 = float(m3.params["FAST_binary"])
    se3 = float(m3.bse["FAST_binary"])
    interaction_p = []
    for term in m3.params.index:
        if "FAST_binary:C(Zone)" in term:
            b = float(m3.params[term])
            s = float(m3.bse[term])
            interaction_p.append(float(2.0 * (1.0 - stats.norm.cdf(abs(b / s)))))
    rows.append(
        {
            "Model": "Linear mixed model, random intercept, FAST x Zone interaction",
            "Estimate": b3,
            "SE": se3,
            "CI95_low": b3 - 1.96 * se3,
            "CI95_high": b3 + 1.96 * se3,
            "p_value": float(2.0 * (1.0 - stats.norm.cdf(abs(b3 / se3)))),
            "Interaction_min_p": float(np.min(interaction_p)) if interaction_p else float("nan"),
        }
    )

    return pd.DataFrame(rows)


def logistic_models(df: pd.DataFrame) -> pd.DataFrame:
    d = df.copy()
    d["FAST_binary"] = np.where(d["FastSlow"] == "FAST", 1, 0)
    d["zMean_0_10_obs"] = zscore(pd.to_numeric(d["Mean_0_10_obs"], errors="coerce"))

    rows = []

    lg1 = smf.logit("FAST_binary ~ zMean_0_10_obs", data=d).fit(disp=False)
    rows.append({"Model": "Logistic model", **logistic_or_ci(lg1, "zMean_0_10_obs")})

    lg2 = smf.logit("FAST_binary ~ zMean_0_10_obs + C(MatchID)", data=d).fit(disp=False)
    rows.append({"Model": "Logistic model, match-adjusted", **logistic_or_ci(lg2,
        "zMean_0_10_obs")})

    lg3 = smf.logit(
        "FAST_binary ~ zMean_0_10_obs + C(Zone) + C(ScoreState) + C(HomeAway) + MinuteMatch",
        data=d,
    ).fit(disp=False)
    rows.append({"Model": "Logistic model, context-adjusted", **logistic_or_ci(lg3,
        "zMean_0_10_obs")})

    return pd.DataFrame(rows)


def logistic_mixed_model(df: pd.DataFrame) -> pd.DataFrame:
    d = df.copy()
    d["FAST_binary"] = np.where(d["FastSlow"] == "FAST", 1, 0)
    d["zMean_0_10_obs"] = zscore(pd.to_numeric(d["Mean_0_10_obs"], errors="coerce"))
    d = d[["FAST_binary", "zMean_0_10_obs", "MatchID"]].dropna().copy()

    model = BinomialBayesMixedGLM.from_formula(
        "FAST_binary ~ zMean_0_10_obs",
        {"Match": "0 + C(MatchID)"},
        d,
```

```
    )
    result = model.fit_map()

    fe_names = list(getattr(result.model, "fep_names", []))
    idx = fe_names.index("zMean_0_10_obs")
    beta = float(np.asarray(result.fe_mean, dtype=float)[idx])
    se = float(np.asarray(result.fe_sd, dtype=float)[idx])
    re_sd = float(np.exp(np.asarray(result.vcp_mean, dtype=float)[0]))
    re_var = float(re_sd**2)

    return pd.DataFrame(
        [
            {
                "Model": "Logistic mixed model",
                "OR": float(np.exp(beta)),
                "CI95_low": float(np.exp(beta - 1.96 * se)),
                "CI95_high": float(np.exp(beta + 1.96 * se)),
                "p_value": float(2.0 * (1.0 - stats.norm.cdf(abs(beta / se)))),
                "RandomInterceptVar_Match": re_var,
                "RandomInterceptSD_Match": re_sd,
                "Converged": "Yes",
            }
        ]
    )


# ---------------------------------------------------------------------------
# One-call wrapper
# ---------------------------------------------------------------------------

def run_all_analyses(df: pd.DataFrame) -> dict[str, pd.DataFrame]:
    """
    Run the set of analyses reported in the manuscript on an analysis-ready
    episode-level dataset.

    This wrapper is intentionally file-agnostic: the dataset-loading step is
    not hard-coded because the corresponding data are not part of the public
    code release.
    """
    return {
        "sample_description": sample_description(df),
        "recovery_time_descriptives": recovery_time_descriptives(df),
        "recovery_time_linear_regression": pd.DataFrame(
            [recovery_time_linear_regression(df)]
        ),
        "primary_comparison": pd.DataFrame([primary_comparison(df)]),
        "zone_stratified_analyses": zone_stratified_analyses(df),
        "match_level_differences": match_level_differences(df),
        "threshold_sensitivity_analyses": threshold_sensitivity_analyses(df),
        "landmark_fixed_window_analyses": landmark_fixed_window_analyses(df),
        "supplementary_table_s1_low_speed": supplementary_table_s1_low_speed(df),
        "supplementary_table_s2_logistic": supplementary_table_s2_logistic(df),
        "linear_models_match_fixed": linear_models_match_fixed(df),
        "linear_mixed_models": linear_mixed_models(df),
        "logistic_models": logistic_models(df),
        "logistic_mixed_model": logistic_mixed_model(df),
    }

# ---------------------------------------------------------------------------
# Mean resultant direction psi_bar_beta and residual diagnostics
# ---------------------------------------------------------------------------
# r_beta = |mean_i exp(j*beta_i)| retains only the MODULUS of the mean complex
# vector. The functions below compute its ARGUMENT (the collective mean
# resultant direction); 0 deg = collective movement toward the goal to be
# defended.

def compute_mean_direction(beta_values):
    """Per-frame circular mean direction psi_bar_beta = arg(mean_i exp(j*beta_i)),
    in radians (-pi, pi]. beta_values: array of the outfield players' beta."""
    beta_values = np.asarray(beta_values, dtype=float)
    beta_values = beta_values[np.isfinite(beta_values)]
    if beta_values.size == 0:
```

```
        return float("nan")
    return float(np.angle(np.mean(np.exp(1j * beta_values))))


def episode_mean_direction(psi_series, recovery_time_s=None,
                           threshold_s=10.0, window_s=None, dt=0.1):
    """Episode-level circular mean of the per-frame direction series over the
    SAME observed window used for Mean_0_10_obs (t_l .. min(recovery, 10 s)),
    or over a fixed window if window_s is given. Returns degrees in (-180, 180];
    0 deg = toward the protected goal."""
    psi = np.asarray(psi_series, dtype=float)
    if window_s is not None:
        n = max(1, int(round(window_s / dt)))
    else:
        end = min(float(recovery_time_s), float(threshold_s))
        n = max(1, int(round(end / dt)))
    psi = psi[:n]
    psi = psi[np.isfinite(psi)]
    if psi.size == 0:
        return float("nan")
    return float(np.degrees(np.angle(np.mean(np.exp(1j * psi)))))


def circular_summary(deg):
    """Circular mean (deg), resultant length R, and % of episodes within
    +/-90 deg of the defended goal, for one group's episode-level directions."""
    deg = np.asarray(deg, dtype=float)
    deg = deg[np.isfinite(deg)]
    rad = np.radians(deg)
    C, S = np.mean(np.cos(rad)), np.mean(np.sin(rad))
    mean_deg = float(np.degrees(np.arctan2(S, C)))
    R = float(np.hypot(C, S))
    toward_goal_pct = float(np.mean(np.abs(deg) <= 90) * 100)
    return len(deg), mean_deg, R, toward_goal_pct


def rayleigh_test(deg):
    """Rayleigh test of uniformity for circular data (degrees).
    Returns (z, p) with the standard small-sample-corrected approximation."""
    deg = np.asarray(deg, dtype=float)
    deg = deg[np.isfinite(deg)]
    n = len(deg)
    rad = np.radians(deg)
    R = np.hypot(np.mean(np.cos(rad)), np.mean(np.sin(rad)))
    z = n * R ** 2
    p = np.exp(-z) * (1 + (2 * z - z ** 2) / (4 * n)
                      - (24 * z - 132 * z ** 2 + 76 * z ** 3 - 9 * z ** 4) / (288 * n ** 2))
    return float(z), float(min(max(p, 0.0), 1.0))


def compare_direction_fast_slow(dir_fast_deg, dir_slow_deg):
    """Group comparison between FAST and SLOW episode-level directions:
    (i) Mann-Whitney U on the absolute angular deviation from 0 deg;
    (ii) chi-square test (1 df, continuity-corrected) on the proportions of
    episodes within +/-90 deg of the defended goal."""
    a = np.abs(np.asarray(dir_fast_deg, dtype=float))
    b = np.abs(np.asarray(dir_slow_deg, dtype=float))
    a, b = a[np.isfinite(a)], b[np.isfinite(b)]
    u_stat, u_p = stats.mannwhitneyu(a, b, alternative="two-sided")
    fa, fb = int(np.sum(a <= 90)), int(np.sum(a > 90))
    sa, sb = int(np.sum(b <= 90)), int(np.sum(b > 90))
    chi2, chi_p, _, _ = stats.chi2_contingency([[fa, fb], [sa, sb]], correction=True)
    return {"mannwhitney_U": float(u_stat), "mannwhitney_p": float(u_p),
            "chi2_1df": float(chi2), "chi2_p": float(chi_p),
            "fast_within90_pct": 100 * fa / (fa + fb),
            "slow_within90_pct": 100 * sa / (sa + sb)}


def lmm_residual_diagnostics(lmm_result, df):
    """Residual diagnostics for the linear mixed model: Shapiro-Wilk on
    residuals, Levene (FAST vs SLOW residual variance) and Breusch-Pagan
    (residuals vs fitted)."""
```

```
    resid = np.asarray(lmm_result.resid, dtype=float)
    fitted = np.asarray(lmm_result.fittedvalues, dtype=float)
    sw_W, sw_p = stats.shapiro(resid)
    lev_W, lev_p = stats.levene(resid[df["FastSlow"] == "FAST"],
                                resid[df["FastSlow"] == "SLOW"])
    import statsmodels.api as sm
    from statsmodels.stats.diagnostic import het_breuschpagan
    bp_lm, bp_p, _, _ = het_breuschpagan(resid, sm.add_constant(fitted))
    return {"shapiro_W": float(sw_W), "shapiro_p": float(sw_p),
            "skewness": float(stats.skew(resid)),
            "kurtosis": float(stats.kurtosis(resid)),
            "levene_W": float(lev_W), "levene_p": float(lev_p),
            "breusch_pagan_LM": float(bp_lm), "breusch_pagan_p": float(bp_p)}


# ---------------------------------------------------------------------------
# Additional reported quantities
# ---------------------------------------------------------------------------


def bootstrap_cohens_d(x: np.ndarray, y: np.ndarray, n_boot: int = N_BOOT,
                       seed: int = BOOT_SEED) -> tuple[float, float]:
    """Percentile bootstrap confidence interval for Cohen's d (FAST vs SLOW)."""
    rng = np.random.default_rng(seed)
    x = np.asarray(x, dtype=float)
    y = np.asarray(y, dtype=float)
    boot = np.empty(n_boot, dtype=float)
    for i in range(n_boot):
        xb = rng.choice(x, size=x.size, replace=True)
        yb = rng.choice(y, size=y.size, replace=True)
        boot[i] = cohens_d(xb, yb)
    return float(np.percentile(boot, 2.5)), float(np.percentile(boot, 97.5))


def auc_from_mann_whitney(x: np.ndarray, y: np.ndarray) -> dict:
    """Rank-based discrimination: AUC = 1 - U/(n1*n2), with the Hanley-McNeil
    standard error and the corresponding 95% confidence interval."""
    x = np.asarray(x, dtype=float)
    y = np.asarray(y, dtype=float)
    n1, n2 = x.size, y.size
    u_stat, _ = stats.mannwhitneyu(x, y, alternative="two-sided")
    auc = 1.0 - float(u_stat) / (n1 * n2)
    q1 = auc / (2.0 - auc)
    q2 = 2.0 * auc ** 2 / (1.0 + auc)
    se = math.sqrt((auc * (1 - auc) + (n1 - 1) * (q1 - auc ** 2)
                    + (n2 - 1) * (q2 - auc ** 2)) / (n1 * n2))
    return {"AUC": auc, "SE": se,
            "CI95_low": auc - 1.96 * se, "CI95_high": auc + 1.96 * se}


def recovery_time_linear_regression(df: pd.DataFrame) -> dict:
    """Simple linear regression of the primary metric on recovery time
    (reported in the Results and in the caption of Figure 7)."""
    d = df[["Mean_0_10_obs", "DeltaRec_s"]].apply(pd.to_numeric,
                                                  errors="coerce").dropna()
    slope, intercept, r_value, p_value, std_err = stats.linregress(
        d["DeltaRec_s"], d["Mean_0_10_obs"])
    n = len(d)
    r2 = r_value ** 2
    adj_r2 = 1 - (1 - r2) * (n - 1) / (n - 2)
    return {"intercept": float(intercept), "slope": float(slope),
            "r_squared": float(r2), "adjusted_r_squared": float(adj_r2),
            "t_value": float(slope / std_err), "df": n - 2,
            "p_value": float(p_value)}


def recovery_time_descriptives(df: pd.DataFrame) -> pd.DataFrame:
    """Median, interquartile range and range of recovery time, reported
    separately for FAST and SLOW episodes."""
    rows = []
    for group in ("FAST", "SLOW"):
        v = pd.to_numeric(df.loc[df["FastSlow"] == group, "DeltaRec_s"],
```

```
                      errors="coerce").dropna()
    rows.append({"Group": group, "n": int(v.size),
                 "Median_s": float(v.median()),
                 "Q1_s": float(v.quantile(0.25)),
                 "Q3_s": float(v.quantile(0.75)),
                 "Min_s": float(v.min()), "Max_s": float(v.max())})
return pd.DataFrame(rows)
```
