# Supplementary material for: Team Synergies in Professional Football: A Goal-Referenced Performance Indicator for Rapid Ball Recovery in Negative Transitions
Source: Sports (Basel). 2026 Sep 1;14(9):374. doi: 10.3390/sports14090374 (PMC13611748; doi:10.3390/sports14090374)
Supplement: Supplementary file 1 [file sports-14-00374-s001.zip › Supplementary_File_S3_Full_statistical_output.pdf]

Supplementary File S3. Full statistical output

This file reports the full statistical output corresponding to the results presented in the manuscript.

1. Sample Description

Matches included: 5
Total episodes: 248
FAST episodes: 133
SLOW episodes: 115
FAST percentage: 53.62903%
SLOW percentage: 46.37097%
Mean recovery time: 13.38 s (SD = 11.31)

Episodes per match

Match 1: 54
Match 2: 44
Match 3: 59
Match 4: 49
Match 5: 42

Zone distribution

Zone 1: 129
Zone 2: 119

Additional descriptive/methodological values reported in the manuscript

Inter-observer agreement on episode validity: 100%
Number of independent UEFA A coaches involved in the video review: 3
Coaching experience of observers: 24.5 ± 2.1 years
Minimum percentage of time spent in the prevalent zone across included episodes: 84%

Landmark sample definition

Episodes recovered within 2 s: 5
Episodes recovered within 3 s: 15
Episodes recovered within 5 s: 53
Therefore:
Landmark 0–2 s: FAST = 128, SLOW = 115
Landmark 0–3 s: FAST = 118, SLOW = 115
Landmark 0–5 s: FAST = 80, SLOW = 115

2. Primary Comparison: Mean_0_10_obs

Descriptive statistics

FAST mean = 0.717392
SLOW mean = 0.599921
Mean difference (FAST − SLOW) = 0.117471
95% CI = [0.084933, 0.150009]
Bootstrap 95% CI = [0.086017, 0.150486]
Cohen’s *d* = 0.906197

Bootstrap 95% CI for d = [0.672664, 1.161079]

Welch *t*-test

t = 7.111891
df = 239.537962
p = 1.31e-11

Mann–Whitney *U* test

U = 4190
p = 8.44e-10
effect size r = 0.389664
Equivalent discrimination: AUC = $1 - U/(n_1 \times n_2)$ = 0.726 (95% CI [0.66, 0.79], Hanley–McNeil)

Mean resultant direction ψβ (FAST vs. SLOW)

mean direction ψβ = arg(Σ $e^{j\beta}$), computed over the same observed window as Mean_0_10_obs; 0° denotes collective movement toward the goal to be defended.
FAST (n = 133): circular mean = −4.8°; R = 0.45; Rayleigh test $p < 0.001$; 78.2% of episodes within ±90° of the defended goal.
SLOW (n = 115): circular mean = −36.9°; R = 0.08; Rayleigh test p = 0.48; 51.3% of episodes within ±90°.
Interpretation: the high rβ of FAST episodes corresponded to coherent collective orientation toward the defended goal (β ≈ 0 mode), whereas SLOW episodes showed no common orientation.
Group comparison: Mann–Whitney U test on the absolute angular deviation from the direction to the defended goal, $p < 0.001$; $\chi^2(1)$ = 18.6, $p < 0.001$ on the proportions of episodes oriented within ±90° of that goal (continuity-corrected).

3. Match-Level FAST–SLOW Differences

Match 1 = 0.125295
Match 2 = 0.208087
Match 3 = 0.103000
Match 4 = 0.099000
Match 5 = 0.105000

4. Table 1 Output: Primary and Zone-Stratified Analyses

Analysis	FAST, *M* (*n*)	SLOW, *M* (*n*)	Mean difference	95% CI	Bootstrap 95% CI	*p*-value	Effect size
Primary comparison 10 s	0.717 (133)	0.600 (115)	0.117	[0.085, 0.150]	[0.086, 0.150]	< 0.001	d = 0.906
Zone 1	0.717 (60)	0.601 (69)	0.116	[0.072, 0.160]	[0.074, 0.160]	< 0.001	d = 0.926
Zone 2	0.718 (73)	0.598 (46)	0.119	[0.068, 0.171]	[0.070, 0.170]	< 0.001	d = 0.877

Exact *p*-values

Primary comparison: 1.31e-11
Zone 1: 6.55e-07
Zone 2: 1.24e-05

5. Table 2 Output: Sensitivity Analyses

Analysis	FAST, *M* (*n*)	SLOW, *M* (*n*)	Mean difference	95% CI	*p*-value	Effect size
Sensitivity threshold 8 s	0.709 (107)	0.628 (141)	0.081	[0.047, 0.115]	< 0.001	*d* = 0.599
Sensitivity threshold 12 s	0.706 (149)	0.598 (99)	0.108	[0.074, 0.142]	< 0.001	*d* = 0.817
Sensitivity threshold 15 s	0.695 (167)	0.597 (81)	0.098	[0.063, 0.134]	< 0.001	*d* = 0.734
Landmark 0–2 s	0.678 (128)	0.564 (115)	0.114	[0.066, 0.162]	< 0.001	*d* = 0.614
Landmark 0–3 s	0.684 (118)	0.569 (115)	0.115	[0.071, 0.159]	< 0.001	*d* = 0.681
Landmark 0–5 s	0.694 (80)	0.587 (115)	0.107	[0.063, 0.151]	< 0.001	*d* = 0.674

Exact *p*-values

Threshold 8 s: 4.21e-06
Threshold 12 s: 1.73e-09
Threshold 15 s: 1.85e-07
Landmark 0–2 s: 4.38e-06
Landmark 0–3 s: 5.13e-07
Landmark 0–5 s: 3.55e-06

6. Table 3 Output: Linear and Logistic Models

Linear models

Match-adjusted linear model (match as fixed effect)

Formula: Mean_0_10_obs ~ FAST + match (fixed effect)
Estimate for FAST = 0.125136
SE = 0.016612
95% CI = [0.092576, 0.157696]
z = 7.532715
p = 4.97e-14
Residual variance = 0.016243

Linear mixed model with random intercept for match

Formula: Mean_0_10_obs ~ FAST + (1|Match)
Estimate for FAST = 0.119776
SE = 0.016971
95% CI = [0.086513, 0.153039]
Random intercept variance for Match = 2.68e-06
ICC for Match = 0.000161 (negligible, not exactly zero)
Note: the random-intercept estimate is close to the raw between-group difference (0.117471), whereas the match-adjusted (fixed-effect) specification recovers the within-match difference (0.125136).

Residual diagnostics (linear mixed model)

Shapiro–Wilk (residuals): W = 0.986, p = 0.017
Residual skewness = −0.294; kurtosis = −0.425
Levene (FAST vs. SLOW residual variance): W = 0.039, p = 0.843

Breusch–Pagan (residuals vs. fitted): LM = 0.065, $p = 0.799$
Interpretation: homoscedasticity supported (Levene, Breusch–Pagan); only a mild, negligible departure from normality.

Context- and match-adjusted linear model (match as fixed effect)

Formula: Mean_0_10_obs ~ FAST + Zone + ScoreState + HomeAway + MinuteMatch + match (fixed effect)
Estimate for FAST = 0.134004
SE = 0.015747
95% CI = [0.103140, 0.164868]
$z = 8.509749$
$p <$ 1e-15 (stored as 0 in the workbook due to numerical formatting)

Interaction linear model (match as fixed effect)

Formula: Mean_0_10_obs ~ FAST * Zone + ScoreState + HomeAway + MinuteMatch + match (fixed effect)
Estimate for FAST in reference zone = 0.125030
SE = 0.021258
95% CI = [0.083364, 0.166696]
$z = 5.881516$
$p =$ 4.07e-09
Minimum interaction p-value = 0.530151

Logistic regression models

Simple logistic model

Formula: FAST ~ zMean_0_10_obs
OR = 2.725421
95% CI = [1.959019, 3.791652]
$p =$ 2.65e-09

Match-adjusted logistic model

Formula: FAST ~ zMean_0_10_obs + C(MatchID)
OR = 3.046304
95% CI = [2.133678, 4.349284]
$p =$ 8.71e-10

Context-adjusted logistic model

Formula: FAST ~ zMean_0_10_obs + Zone + ScoreState + HomeAway + MinuteMatch
OR = 3.696178
95% CI = [2.501018, 5.462467]
$p =$ 5.38e-11

Supplementary logistic mixed model

Formula: FAST ~ zMean_0_10_obs + (1|Match)
OR = 2.819556
95% CI = [1.998026, 3.978875]
$p =$ 3.66e-09

Random intercept variance for Match = 0.138152
Random intercept SD for Match = 0.371688
Converged = Yes
Estimation: Bayesian maximum a posteriori (BinomialBayesMixedGLM); OR, CI and *p*-value are Wald-type approximations around the posterior mode.

7. Full Output for Supplementary Table S1: Low-Speed Technical Sensitivity

Summary table aligned with the manuscript supplementary material

Technical check	Velocity threshold	Low-speed handling	Low-speed player-samples, %	Summary of result
Alternative threshold 1	0.20 m/s	Circular interpolation	3.21	FAST > SLOW direction preserved; $p < 0.001$
Alternative threshold 2	0.50 m/s	Circular interpolation	5.38	FAST > SLOW direction preserved; $p < 0.001$
Alternative threshold 3	0.70 m/s	Circular interpolation	7.74	FAST > SLOW direction preserved; $p < 0.001$
Alternative handling 1	0.35 m/s	Exclusion below threshold	4.15	FAST > SLOW direction preserved; $p < 0.001$
Alternative handling 2	0.35 m/s	Speed-weighted rβ	4.15	FAST > SLOW direction preserved; $p < 0.001$

Exact *p*-values

0.20 m/s, circular interpolation: 8.09e-13
0.50 m/s, circular interpolation: 6.20e-13
0.70 m/s, circular interpolation: 9.95e-13
0.35 m/s, exclusion below threshold: 2.07e-14
0.35 m/s, speed-weighted rβ: 2.77e-12

8. Full Output for Supplementary Table S2: Simple Logistic Models for Sensitivity Metrics

Analysis	Logistic model	Predictor	OR	95% CI	*p*-value
Threshold 8 s	FAST8 ~ zMean_0_8_obs	zMean_0_8_obs	1.95	[1.46, 2.61]	< 0.001
Threshold 12 s	FAST12 ~ zMean_0_12_obs	zMean_0_12_obs	2.31	[1.69, 3.16]	< 0.001
Threshold 15 s	FAST15 ~ zMean_0_15_obs	zMean_0_15_obs	1.95	[1.45, 2.63]	< 0.001
Landmark 0–2 s	FAST_landmark2 ~ zMean_0_2_landmark	zMean_0_2_landmark	1.88	[1.42, 2.49]	< 0.001
Landmark 0–3 s	FAST_landmark3 ~ zMean_0_3_landmark	zMean_0_3_landmark	2.04	[1.52, 2.74]	< 0.001
Landmark 0–5 s	FAST_landmark5 ~ zMean_0_5_landmark	zMean_0_5_landmark	2.04	[1.46, 2.83]	< 0.001

Exact *p*-values

Threshold 8 s: 7.37e-06
Threshold 12 s: 1.43e-07
Threshold 15 s: 1.10e-05
Landmark 0–2 s: 9.33e-06
Landmark 0–3 s: 2.42e-06
Landmark 0–5 s: 2.34e-05
