# Supplementary material for: Team Synergies in Professional Football: A Goal-Referenced Performance Indicator for Rapid Ball Recovery in Negative Transitions
Source: Sports (Basel). 2026 Sep 1;14(9):374. doi: 10.3390/sports14090374 (PMC13611748; doi:10.3390/sports14090374)
Supplement: Supplementary file 1 [file sports-14-00374-s001.zip › Supplementary_File_S4_Completed_STROBE_checklist.pdf]

Supplementary File S4. Completed STROBE Checklist

STROBE item	Location in manuscript
**1a. Study design in title or abstract**	Abstract
**1b. Informative and balanced summary of methods and findings**	Abstract
**2. Scientific background and rationale**	Introduction
**3. Specific objectives and hypotheses**	Introduction, final paragraph
**4. Key elements of study design**	Materials and Methods – Study design and sample
**5. Setting, locations, dates, and data collection period**	Materials and Methods – Study design and sample (start of the 2024/2025 season)
**6a. Eligibility criteria and selection of participants/episodes**	Materials and Methods – Study design and sample; Episode identification and outcome definition
**6b. Matching criteria and matched groups**	N/A. No matching procedure was used.
**7. Outcomes, exposures, predictors, potential confounders, and effect modifiers**	Materials and Methods – Episode identification and outcome definition; Computation of rβ; Contextual variables; Statistical analysis
**8. Data sources and measurement procedures**	Materials and Methods – Positional and video data; Computation of rβ
**9. Efforts to address potential sources of bias**	Materials and Methods – Positional and video data; Episode identification and outcome definition; Contextual variables; Discussion – Limitations and future directions
**10. Study size**	Materials and Methods – Study design and sample
**11. Handling of quantitative variables**	Materials and Methods – Computation of rβ; Time-windowing strategy and sensitivity analyses; Contextual variables
**12a. Statistical methods, including methods used to control for confounding**	Materials and Methods – Statistical analysis; Complementary linear and logistic models
**12b. Subgroup and interaction analyses**	Materials and Methods – Statistical analysis; Complementary linear and logistic models
**12c. Missing data**	Materials and Methods – Statistical analysis
**12d. Loss to follow-up, matching, or sampling strategy**	N/A. This was an event-based observational study without participant follow-up, matching, or survey sampling.
**12e. Sensitivity analyses**	Materials and Methods – Time-windowing strategy and sensitivity analyses; Statistical analysis – Sensitivity comparisons
**13a. Number of episodes at each stage**	Materials and Methods – Study design and sample; Episode identification and outcome definition; Contextual variables (345 sequences identified → 97 excluded → 248 analysed; 133 FAST, 115 SLOW)

**13b. Reasons for exclusion/non-participation**	Materials and Methods – Episode identification and outcome definition (97 of 345 sequences excluded per the predefined criteria)
**13c. Flow diagram**	Not included. Numbers at each stage reported in Materials and Methods – Episode identification and outcome definition (345 → 97 → 248).
**14a. Descriptive characteristics of the analysed episodes**	Materials and Methods – Study design and sample; Episode identification and outcome definition; Contextual variables. Descriptive information includes FAST/SLOW counts, mean recovery time, match distribution, field-zone distribution, and availability of contextual covariates.
**14b. Missing data for each variable of interest**	Materials and Methods – Statistical analysis
**14c. Follow-up time**	N/A. No participant follow-up was conducted.
**15. Outcome data**	Results – Main FAST–SLOW comparison
**16a. Main unadjusted and adjusted estimates with precision**	Results – Main FAST–SLOW comparison; Linear and logistic regression models; Tables 1 and 3
**16b. Category boundaries for categorized variables**	Materials and Methods – Episode identification and outcome definition; Time-windowing strategy and sensitivity analyses; Contextual variables
**16c. Translation of relative risk into absolute risk**	N/A. The study did not estimate relative risk over a follow-up period.
**17. Other analyses, including subgroup, interaction, and sensitivity analyses**	Results – Sensitivity analyses; Linear and logistic regression models; Tables 2 and 3
**18. Summary of key results**	Discussion, opening paragraph
**19. Limitations and potential sources of bias or imprecision**	Discussion – Limitations and future directions
**20. Cautious overall interpretation**	Discussion; Conclusions
**21. Generalisability / external validity**	Discussion – Limitations and future directions
**22. Funding source and role of funders**	Funding details
